# Occurrence and Dietary Exposure to Acrylamide from Foods Consumed within and outside Main Meals in Singapore

**DOI:** 10.3390/foods12163022

**Published:** 2023-08-11

**Authors:** Wesley Zongrong Yu, Ping Shen, Ignatius Lim, Raymond Rong Sheng Shi, Miaohua Cai, Yee Soon Chin, Ai Jin Tay, Wei Min Ang, Jun Cheng Er, Geraldine Songlen Lim, Yuansheng Wu, Angela Li, Kyaw Thu Aung, Sheot Harn Chan

**Affiliations:** 1 National Centre for Food Science, Singapore Food Agency, 7 International Business Park, Singapore 609919, Singapore; wesley_yu@sfa.gov.sg (W.Z.Y.);; 2 School of Biological Sciences, Nanyang Technological University, 60 Nanyang Drive, Singapore 637551, Singapore; 3 Department of Food Science and Technology, 2 Science Drive 2, Faculty of Science, National University of Singapore, Singapore 117543, Singapore

**Keywords:** acrylamide, foods, snack, LC-MS/MS, dietary exposure, risk assessment, MOE, Singapore

## Abstract

This study investigated the influence of ‘snackification’ in Singaporean diets, leading to increased dietary acrylamide exposure. Acrylamide concentrations in commonly consumed foods within and outside the main meals were measured using liquid chromatography with tandem mass spectrometry (LC-MS/MS). High acrylamide concentrations were detected in vegetables cooked at high temperatures (ranging from 0.5 to 478.4 µg/kg) and potato-based crackers and chips (ranging from 81.8 to 2095.8 µg/kg). The estimated total dietary exposure for the Singapore population was 0.165 µg/kg bw/day for general consumers and 0.392 µg/kg bw/day for high consumers (95th percentile). The acrylamide exposure from outside main meals was nearly equivalent to that from within the main meals. The calculated margins of exposure (MOE) were below 10,000, indicating potential human health concern. These findings highlight the need for industry practices and consumer advisories to reduce acrylamide exposure from foods consumed both within and outside main meals.

## 1. Introduction

Acrylamide, classified as a probable carcinogen to humans (Group 2A) by the International Agency for Research on Cancer (IARC) [1], has been extensively studied for the past two decades since its seminal discovery in heat-processed food by Swedish researchers in 2002 [2]. Research has focused on determining its occurrence levels in foods, investigating the factors and mechanisms leading to its formation, and conducting toxicology studies [3]. Acrylamide primarily forms through the Maillard reaction between reducing sugars and specific amino acids such as asparagine, present in carbohydrate-rich foods [4,5]. Foods like fried potatoes, biscuits, bread, roasted coffee, and other starchy heat-processed products have been widely reported to contain higher levels of acrylamide. However, significant variability in acrylamide concentrations has been reported, even within the same type of foods [6,7]. This variability is attributed, in part, to factors such as the ingredient composition, carbohydrate levels, free asparagine content, type of reducing sugars, pH, water content, cooking temperature, and duration.

In response to concerns about acrylamide, the Food Agriculture Organisation (FAO)/World Health Organisation (WHO) Joint Expert Committee on Food Additives (JECFA) conducted multiple rounds of exposure and risk assessment in 2002, 2005, and 2011 [8,9,10]. Their 2011 assessment, based on data compiled from 27 countries (with 66% of the data from Europe), revealed that the average dietary exposure of acrylamide was from 1.1 µg/kg body weight (bw)/day for the general population to 4.8 µg/kg bw/day for high consumers [11]. JECFA concluded that these dietary exposures were comparable to the 2005 assessment and raised human health concerns. In light of these findings, the FAO/WHO Codex Alimentarius developed a ‘Code of Practice for the Reduction of Acrylamide in Foods’ (CAC/RCP 67-2009) to provide guidance for national food authorities and agencies, manufacturers, and relevant bodies [12]. However, it is important to note that there are currently no international regulatory limits set for acrylamide in food.

National food authorities and agencies worldwide have implemented country-specific programs to monitor acrylamide levels in foods, conduct risk assessments, estimate dietary exposure, and establish risk mitigation measures. In 2015, the European Food Safety Authority (EFSA) published its scientific opinion on acrylamide in food, reporting mean and 95th percentile dietary acrylamide exposures ranging from 0.4 to 1.9 µg/kg bw/day and 0.6 to 3.4 µg/kg bw/day, respectively [13]. EFSA concluded that these dietary exposure levels raised concerns about neoplastic effects based on animal evidence. The highest acrylamide levels were found in solid coffee substitutes and coffee and fried potato products. In 2018, the European Union (EU) established mitigation measures and benchmark levels to guide the industry in reducing acrylamide in certain food categories [14]. In the United States (US), the US Food and Drug Administration (FDA) monitored acrylamide levels in foods during two different time periods (2002–2006 and 2011–2015) [15,16]. Significant decreases in acrylamide concentrations were observed in potato chips and crackers, providing evidence that the industry had adopted mitigation strategies. The main contributors to acrylamide exposure in the US were still French fries, breakfast cereal, cookies, potato chips, and crackers in both time periods. The mean dietary acrylamide exposures for Americans aged 2 years and older were 0.44 µg/kg bw/day (2002–2006) and 0.36 µg/kg bw/day (2011–2015). In comparison, the dietary exposures for Americans were slightly lower than those for Europeans.

The makeup of Asian diets differs significantly from those of Europeans and Americans, leading to lower reported dietary acrylamide exposures in Asian populations [6]. For example, the first Hong Kong Total Diet Study in 2014 reported mean dietary acrylamide exposures of 0.213 µg/kg bw/day for the general population and 0.538 µg/kg bw/day for high consumers (95th percentile) [17]. The sixth Chinese TDS (2016–2019) reported a mean exposure of 0.175 µg/kg bw/day [18]. The Food Safety Commission of Japan (FSCJ) reported a point estimate of 0.240 µg/kg bw/day for Japanese people in 2016 [19]. Additionally, the Korean National Health and Nutrition Examination Survey (2013–2017) reported the lowest exposure among Asian countries at 0.09 µg/kg bw/day [20]. In these studies, the highest acrylamide concentrations were found in snack foods (mean value of 680 µg/kg) in Hong Kong, potato samples (211.8 µg/kg) in China, molded mashed potato snacks (1187 µg/kg), and teas made of Jerusalem artichoke (7331.0 µg/kg) in Korea. It should be noted that, unlike the Europeans and Americans, vegetables cooked at high temperatures were among the main contributors to the overall estimated acrylamide exposure in China, Japan, and Hong Kong, whereas confectioneries and coffee were the main contributors in Korea. Despite these studies in Asia, information on the occurrence and dietary exposure of acrylamide in foods in Singapore remains scarce, except for one study conducted in 2021 on the acrylamide content in 30 local snacks [21].

The necessity of country-specific studies on the dietary exposure of acrylamide becomes apparent when considering the widespread occurrence levels and significant variation of acrylamide in food, as well as the diverse diets across different populations [6,7]. In this study, we present the occurrence levels and dietary exposure of acrylamide in commonly consumed foods by the general population (aged 15 to 92 years old) in Singapore. Notably, our study distinguished between foods consumed within and outside the three main meals (breakfast, lunch, dinner) in typical Singaporean diets. To the best of our knowledge, this is the first study to investigate dietary acrylamide exposure with a specific focus on the rising ‘snackification’ trends in the Asia-Pacific region [22,23,24]. While certain foods like fried potatoes, biscuits, cereal-based snacks, and roasted coffee are traditionally more prevalent in European and American diets, they have gained popularity in Singapore and other developed countries due to the increasing trend of snacking. Hence, it is essential to examine the contribution of these snack foods consumed outside of main meals to the overall dietary exposure of acrylamide. Our study aims to elucidate the contribution from snacking foods on acrylamide exposure and provide valuable insights for targeted industry practices and consumer advisories. By understanding the role of foods consumed both within and outside main meals, we can effectively reduce dietary exposure of acrylamide and promote better health outcomes.

## 2. Materials and Methods

### 2.1. Chemicals and Reagents

Standard acrylamide was obtained from ChemService (Westchester, PA, USA). Deuterated reference standard of acrylamide-2,3,3-d_3_ used as internal standard was obtained from CDN Isotopes (Point-Claire, QC, Canada). All standards were of ≥97% purity. All solvents used in the study were of HPLC grade and above, except for *n*-hexane, with a residue grade. Acetonitrile (MeCN) was from Anhui Fulltime Specialized Solvents and Reagents Co., Ltd. (Anqing, China), *n*-hexane was from Tedia (Fairfield, CT, USA), and methanol (MeOH) was from EAM (Selangor, Malaysia). Anhydrous magnesium sulphate (MgSO_4_), AR grade was obtained from Nacalai Tesque (Kyoto, Japan), and sodium chloride (NaCl), AR grade was from VWR International, LLC (Leuven, Belgium). Formic acid (≥98%), AR grade was from Sinopharm Chemical Reagent Co., Ltd. (Shenyang, China). Ultrapure water with a specific resistance of 18.2 MΩ-cm, was produced by an ultrapure water system, PURELAB Option-Q (Veolia, High Wycombe, UK).

### 2.2. Preparation of Standard Solutions

Calibration standard stock solution and isotope-labeled standard stock solution were prepared by dissolving acrylamide and acrylamide-2,3,3-d_3_ (both approximately 10 mg), respectively, in methanol (10 mL) using volumetric flasks. The working standard solutions (100 and 1000 µg/kg) were freshly prepared on the day of the experiments by serial dilution from intermediate standard solution at 1 × 10^4^ µg/kg with ultrapure water. The working internal standard solution (1000 µg/kg) was also freshly prepared on the day of the experiments by serial dilution from isotope-labeled standard intermediate solutions at 1 × 10^4^ µg/kg with ultrapure water. The stock solutions were stored at −4 °C in a fridge for at most one month.

### 2.3. Sample Collection and Preparation

Foods commonly consumed by the population in Singapore were determined from food consumption surveys by the Singapore Food Agency (SFA) and National Nutrition Survey (Singapore) by the Health Promotion Board (HPB) of Singapore [25].

For the main meals, a total of 13 food categories were identified with 261 food products commonly consumed in Singapore with different cooking methods (Supplementary Materials, Tables S1–S19). The food categories consisted of vegetables (7 different types), vegetable protein, fruit and fruit products, grain and grain-based products, meat and meat products, eggs and egg products, milk and dairy products, fish and seafood, fats and oils, sauces and condiments, composite foods, beverages (excluding coffee and coffee substitutes products), and bakery products (excluding biscuits and wafers). A competent team of consultants with decades of culinary experience as chefs was engaged to perform the food preparation and cooking of the food samples. The consultants also provided advice on consumers’ preferences and habits in food preparation, which were adopted in this study. For example, meats were predominantly diced into cubes, fish were sliced, and vegetables were chopped into smaller pieces. There were 14 different cooking methods (bake, boil, braise, brew, deep fry, grill, half-boil, pan fry, reconstitute, roast, stir fry, steam, soup, and stew) including ready-to-eat and no cooking. Table 1 shows the main cooking methods and temperatures. The foods sampled were homogenised and kept in refrigerator at −4 °C until analysis. A total of 385 food samples were analysed for their concentrations of acrylamide.

For foods consumed outside the main meals, a total of 3 food categories were identified with 386 food samples purchased from local supermarkets and online platforms. The food categories consisted of ready-to-eat savouries (crackers and chips based on main ingredients, biscuits and wafers, cereal-based snack bars, and dried snack foods), beverages (roasted coffee, instant coffee, coffee substitute exclusively from cereals), and nuts (almonds, Brazil nut kernels, cashew, chestnuts, peanuts, pistachios, walnuts, and mixed nuts).

### 2.4. Standard Addition Procedure and Method Validation

Standard addition with linear regression calibration was used for the measurement of acrylamide in food samples. Six calibration solutions were prepared at two different concentration ranges—low (1 to 200 µg/kg) and high (50 to 1000 µg/kg)—by pipetting appropriate amount of acrylamide working standard solutions (100, 1000, and 10,000 µg/kg) and working internal standard solution (1000 µg/kg) into a suitable matrix-matched material. Quality control materials were also prepared at 100 µg/kg (low) or 400 µg/kg (high) by adding 100 µL or 400 µL of acrylamide standard solution (1000 µg/kg) and 200 µL of internal standard solution (1000 µg/kg) into a suitable matrix-matched material. The recoveries for the quality control materials should be between 80% and 120%. The isotope mass ratio obtained from weighing and the measured isotope ratio by LC-MS/MS analysis should have a linear relationship. A calibration curve with a goodness-of-fit for linear regression (R^2^) better or equal to 0.99 was accepted for calculation of acrylamide concentration.

The method was validated at 100 µg/kg and 500 µg/kg in both prawn cracker and bread samples with spike recovery within 80 to 120%. The method precision was evaluated under repeatability conditions with 3 replicates. The relative standard deviations were all less than 5%, indicating good method repeatability. The limit of detection (LOD) and limit of quantitation (LOQ) were estimated to be 1 µg/kg and 3 µg/kg, respectively, by spiking into blank samples in 7 replicates. Our method was further validated by participation in proficiency testing programme (FAPAS) provided by Fera Science Ltd. (FERA). Our results were found to be satisfactory (z-score < 2) for the analysis of acrylamide in both biscuits (cookies) (FAPAS 30126) and coffee (instant) (FAPAS 3097). This demonstrated the robustness and comparability of our method with other participating international testing laboratories.

### 2.5. Preparation of Sample for Acrylamide Analysis

Approximately 1 g of homogenised sample was added into a 50 mL polypropylene conical tube and was spiked with 200 µL of internal standard solution (1000 µg/kg) and 800 µL of ultrapure water. The spiked sample was vortexed and left to equilibrate for 15 min. Afterward, 5 mL of *n*-hexane was added to the sample and vortexed. Vortex mixing was repeated with the sequential addition of 10 mL acetonitrile followed by 10 mL of ultrapure water. The mixture was combined with a pre-weighed QuEChERS salt mixture (4 g of MgSO_4_ and 0.5 g of NaCl) and was vortexed for 1 min and centrifuged at 4000 rpm for 10 min. The 5 mL acetonitrile layer was transferred into a 15 mL polypropylene conical tube and dried completely using a stream of nitrogen at 45 °C. The sample extract was then reconstituted in 500 µL ultrapure water, filtered through a 0.2 µm nylon filter, and submitted to LC-MS/MS analysis.

### 2.6. LC-MS/MS Analysis

The LC-MS/MS analysis was performed on an Agilent 1290 Infinity LC system (Agilent Technologies, Waldbronn, Germany) coupled with a Qtrap 5500 quadrupole tandem mass spectrometer (Sciex, Singapore) equipped with an electrospray ionization (ESI) source. The multiple reaction monitoring (MRM) mode was used for the detection of acrylamide. The chromatographic separation was performed on the LC with porous graphitic carbon column (Hypercarb^TM^ Porous Graphitic Carbon HPLC, 100 mm × 3.0 mm i.d., 5 µm, ThermoFisher Scientific, Waltham, MA, USA). Secondary LC columns (Zorbax SB-CN, 100 mm × 3.0 mm i.d., 3.5 µm, Agilent Technologies, (Santa Clara, CA, USA) and/or Zorbax SB-Phenyl, 100 mm × 3.0 mm i.d., 3.5 µm, Agilent Technologies, (Santa Clara, CA, USA) were used to provide additional verification of the sample when it was detected on the primary column. The mobile phase used for both primary and secondary columns consisted of ultrapure water with 0.001% formic acid (Eluent A) and methanol with 0.001% formic acid (Eluent B). The flow rate was 0.3 mL/min, and the isocratic program was as follows: the percentages of eluents A and B were 90% and 10%, respectively. The column temperature was 50 °C, and injection volume was 10 µL.

The MS/MS detection was operated under positive ESI mode. The MS parameter settings such as precursor and product ions, and collision energies are presented in Table 2. Operating conditions for ESI were as follows: IonSpray voltage (IS): 4500 V (+), ion source temperature at 400 °C, curtain gas (CUR) pressure of 30 psi, ion source gas 1 (GS1) and ion source gas 2 (GS2) pressures of 60 psi.

### 2.7. Statistical Analysis of Acrylamide Concentrations and Food Consumption Data

A total of 16 food categories were studied (Supplementary Materials, Tables S1–S22). The acrylamide concentration of each food samples was measured by LC-MS/MS. It should be noted that concentrations below the LOD (1 µg/kg), known as left-censored (LC) data, were substituted with constant values following WHO recommendations on the evaluation of low-level contaminant of food [26]. The substitution method was applied by EFSA and is essential for the estimation of dietary exposure assessment [13,27]. The concentration values for those food samples below LOD were set at 0 µg/kg when the food category contains more than 60% left-censored data (i.e., LOD < 1 µg/kg). On the other hand, the concentration values for those food samples below LOD were set at half of the LOD (0.5 µg/kg) when the food category contains less than 60% left-censored (i.e., LOD < 1 µg/kg) data. Other statistical analysis such as median, standard deviation of the mean (SD mean), and coefficient of variation (CV) were calculated.

The food consumption data of the general consumers and high consumers (95th percentile) in the Singapore population were obtained from 24-h dietary recall surveys conducted in 2021–2022 with participants aged 15 to 92 years old. The mean consumption amount in each food product was based on the averaging the consumption amount reported by individual respondents.

### 2.8. Dietary Exposure Assessment and Risk Assessment

The estimation of dietary exposure to acrylamide was performed by combining data on acrylamide concentrations and food consumption. Although a probabilistic approach is known to obtain the best estimate of dietary exposure [28], in many cases, a deterministic approach as a screening method is appropriate to ascertain risk [29,30]. In this study, a deterministic approach was selected to estimate the dietary exposure to acrylamide of an individual consumer (µg/kg bw/day) (Equation (1)):

(1) $$Dietary\;Exposure=\sum_{j=1}^{N}\frac{\sum_{i=1}^{n}C_{i}\times E_{i}}{n}$$

where *C* is the acrylamide concentration in each food samples (µg/kg) denoted as *i*, and *E* is the mean consumption data in each food samples per kilogram body weight of an individual (kg/kg bw day), which are point estimates used for the dietary exposure calculation for each food category. *n* is the number of food samples in each food category, and *N* is the number of individual food categories denoted as *j*.

Risk assessment tools such as margins of exposure (*MOE*), target hazard quotient (THQ), and incremental lifetime cancer risk (ILCR) could be employed to ascertain the risk from dietary acrylamide [31,32,33,34,35]. Our study utilised MOE, which was also the tool used by JECFA and EFSA in their risk assessment of dietary acrylamide. The lower limit on the BMD for a 10% response (*BMDL*_10_) in toxicology studies of laboratory animals was used to derive the reference points from which the margin of exposure (*MOE*) can be calculated (Equation (2)). An MOE of 10,000 (based on *BMDL*_10_) or higher indicates low level of safety concern that does not constitute health risk for genotoxic compounds [31].

(2) $$MOE=\frac{{BMDL}_{10}}{Dietary\;exposure}$$

## 3. Results and Discussion

### 3.1. Foods Consumed within the Three Main Meals of an Asian Diet in Singapore

A traditional Asian diet typically emphasises a wide variety of whole and minimally processed foods, such as vegetables, legumes, roots and tubers, grains, fish, lean meats, and fruits [36,37]. This diet is rich in fiber and plant-based proteins and commonly includes the use of sauces and condiments during cooking. The Singaporean diet, influenced by Chinese, Malay, and Indian culinary traditions, is a fusion of multicultural flavours and also includes portions of eggs, dairy, and bakery products. Table 3 summarised the acrylamide levels in various food categories commonly found in the Singaporean diet. A total of 385 food samples were analysed for acrylamide concentrations, with a positive detection rate of 32.5% (126 out of 385 samples). Our study revealed that the mean acrylamide concentrations were highest in the following order: sauces and condiments (mean ± std dev., 38.8 ± 19.5 µg/kg), vegetables (32.2 ± 7.6 µg/kg), and composite foods (17.7 ± 7.1 µg/kg). The remaining food categories in Table 3 were found with lower mean acrylamide concentrations, all below 10 µg/kg. Notably, the majority of food categories did not show detectable levels of acrylamide. Among them, the percentage of left-censored (LC) results, where acrylamide was below the LOD, ranked as follows: fats and oils (100%), fruits and fruit products (95.3%), milk and dairy products (93.3%), eggs and egg products (90.9%), fish and seafood (83.9%), vegetable protein (83.3%), grain and grain-based products (65.4%), and meat and meat products (57.8%).

**Figure 1:**
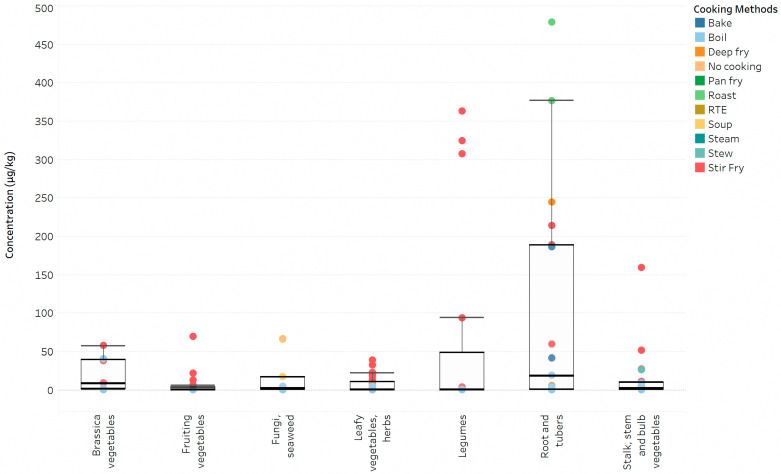
The box-and-whisker plots of different types of vegetables where their acrylamide concentrations and cooking methods are represented by a circle and respective colours. The interquartile ranges (IQR) were illustrated by the boxes where they were bounded by the lower quartile (Q1) (i.e., 25th percentile of data) and the upper quartile (Q3) (i.e., 75th percentile of data). The minimum (i.e., Q1 − 1.5 × IQR) and maximum (i.e., Q1 + 1.5 × IQR) values were represented by the whisker lines. Outliers were represented by values found outside of the box-and-whisker plots.

In this study, we examined various cooking methods used for vegetables, including brassica, fruiting, leafy and herbs, legumes, stalks, stem, and bulb, root and tubers, fungi, and seaweed (Table 3). Acrylamide concentrations ranged from 0.5 to 478.4 µg/kg, with 45.8% of the vegetable samples (55 out of 120) tested positive for acrylamide. The large coefficients of variation (CV) within each vegetable food category, like 112.1% for brassica and 241.7% for fruiting vegetables, highlighted the influence of vegetable varieties and cooking methods on acrylamide formation. While previous studies from Hong Kong, Japan, and China identified vegetables cooked at high temperatures as major food contributors to dietary acrylamide in their populations [17,18,19], the specific effects of cooking methods (temperature and moisture) on acrylamide formation have been less studied. Figure 1 illustrates the distribution of acrylamide concentrations based on vegetable types and cooking methods. Stir-frying at 150 °C resulted in 80% positive samples (32 out of 40) with acrylamide concentrations ranging from 3.3 to 362.9 µg/kg. Some stir-fried legumes (pea, pea sprout, beansprout), roots and tubers (lotus root, potato), and stem (ginger) showed higher acrylamide levels (159.0 to 362.9 µg/kg), comparable to findings in a Hong Kong study (140 to 360 µg/kg) [17]. Other cooking methods with high temperatures, such as baking (180 °C), deep frying (180 °C), and roasting (200 °C), also resulted in high acrylamide concentrations, ranging from 185.9 to 478.4 µg/kg. However, these higher levels were primarily found in root vegetables and tubers (potato and sweet potato), which are known to contain abundant acrylamide precursors, namely reducing sugars and asparagine amino acids. Steamed root vegetables and tubers (sweet potato, yam) and fruiting vegetables (brinjal) had undetectable acrylamide levels. Steamed stems (ginger) and stewed bulb vegetables (garlic) showed low acrylamide concentrations at 4.9 µg/kg and 26.4 µg/kg, respectively. Boiled vegetable samples (100 °C) had a lower detection rate at 19% (9 out of 48) with acrylamide concentrations ranging from 1.8 to 40.4 µg/kg. An independent two samples *t*-test on the mean acrylamide concentrations of stir-fried (55.5 µg/kg) and boiled (2.4 µg/kg) vegetable samples revealed a significant difference (*p* = 0.0002 < 0.05), showing evidence of the influence of cooking temperatures on acrylamide levels.

### 3.2. Ready-to-Eat Snack Foods Consumed outside the Three Main Meals in Singapore

Singapore, like other Asia Pacific countries, witnessed a rise in the ’snackification‘ trend due to urbanisation, leading to changes in eating habits towards processed and convenient snack foods [22,23]. These include potato chips, crackers, biscuits, instant coffee, pastries, and savouries, which are often consumed outside the main meals. In this study, we focused on studying the acrylamide levels in these snack foods and analysed a total of 386 samples, including ready-to-eat savories, beverages (coffee), and nuts. The positive detection rate was 80.1%, significantly higher than the 32.5% for main meals, indicating the need to investigate acrylamide intake from snacks for exposure assessment. Among the snack products summarised in Table 4, ready-to-eat (RTE) savouries like crackers and chips had the highest mean acrylamide concentration (mean ± std dev., 249.5 ± 36.2 µg/kg), while nuts were found to be the lowest (41.4 ± 8.1 µg/kg).

Crackers and chips obtain their crispy and crunchy textures through deep frying or baking at high temperatures, which leads to the removal of moisture. These cooking conditions play a significant role in the formation of acrylamide, particularly when the main ingredients contain high content of acrylamide precursors such as asparagine and reducing sugar [39,40]. The crackers and chips examined in this study were divided into six sub-categories based on their main ingredients. Potato-based products showed the highest mean acrylamide concentration at 732.7 µg/kg. On the other hand, the five remaining sub-categories (fruits, wheat flour, rice flour, vegetables, and seafood-based) consisted of non-potato-based products with lower acrylamide levels, as they contain little or no acrylamide precursors. It should be noted that 41.7% (15 out of 36) of potato-based products exceeded the EU benchmark level of 750 µg/kg for potato-based crackers [14]. Similar findings were reported in recent studies conducted in Romania and Bangladesh [41,42]. Although there was also evidence of reduced acrylamide levels in potato crisps in Europe [43], risk mitigation measures such as reducing acrylamide formation during food processing remain ongoing research areas [44,45].

Other RTE savory snacks examined in our study included biscuits and wafers, cereal-based snack bars, and popcorns. These snacks are usually baked or roasted at moderate temperatures. The mean acrylamide concentrations were 195.7 µg/kg for biscuits and wafers, and 126.9 µg/kg for cereal-based snack bars and popcorns. The formation of acrylamide is mainly attributed to the combination of free asparagine in wheat flour and cereals with reduced sugars during their cooking process [46,47]. Notably, acrylamide was detected with a high positive occurrence rate of over 90% in these snacks. In contrast, dried seafood-based snacks had a lower positive occurrence rate of 38.2%, with the mean concentration of 11.8 µg/kg being the lowest among all the RTE savory snacks.

Coffee derives its appealing aroma and flavour from high-temperature roasting, grinding, and brewing. These processes, along with naturally present asparagine and reducing sugars in coffee beans, contribute to acrylamide formation [48,49,50]. In our study, roasted coffee had a mean acrylamide concentration of 146.0 µg/kg, while instant coffee had 60.4 µg/kg, and cereal-based coffee substitute had 75.8 µg/kg. Interestingly, another study reported higher mean acrylamide concentrations for coffee substitutes (818 µg/kg), followed by instant coffee (358 µg/kg) and then roasted coffee (179 µg/kg) [51]. While this observation remains unclear, our findings of lower acrylamide levels in instant coffee could potentially be explained by the acrylamide loss during the additional freeze-drying process used in instant coffee production. Additionally, the acrylamide levels in our study were lower and below the respective EU benchmark levels.

Nuts are nutrient-rich, energy-dense foods with beneficial unsaturated fatty acids. However, they also contain acrylamide precursors like asparagine and reducing sugars, which differ across nut varieties [52]. Acrylamide primarily forms during roasting in nut processing. The nuts examined in this study were grouped into eight sub-categories based on their types. Mixed nuts had the highest mean concentration of acrylamide at 75.7 µg/kg. These are mostly almond-based, which is consistent with reports of almonds being richer in asparagine and sucrose contents [52]. Other nut varieties like chestnuts, peanuts, cashew, and walnuts contained lower mean acrylamide concentrations, (19.7 to 46.0 µg/kg).

### 3.3. Estimation of the Dietary Exposure from Foods Consumed within and outside the Main Meals

Our study aimed to understand the dietary exposure of acrylamide in the Singaporean diet by comparing contributions from foods consumed within and outside main meals (Table 5). For general consumers, the estimated dietary exposure of acrylamide within meal meals was 42.6% at 0.070 µg/kg bw/day, while that outside of main meals was 57.4% at 0.095 µg/kg bw/day. The total dietary exposure of acrylamide for general consumers was estimated to be 0.165 µg/kg bw/day. For high consumers (95th percentile), the estimated dietary exposure of acrylamide within meal meals was 45.4% at 0.178 µg/kg bw/day, while that outside of main meals was 54.6% at 0.214 µg/kg bw/day. The total dietary exposure of acrylamide for high consumers was estimated to be 0.392 µg/kg bw/day. The contributions of individual food categories to the total dietary exposure of acrylamide for general and high consumers were plotted in the Supplementary Materials as Figures S1 and S2, respectively.

It is worth noting that the contributions from foods consumed within and outside main meals were nearly equivalent. Similar findings were reported by Jeong et. al. on the Korean population, where confectioneries and coffee were the main contributors to acrylamide exposure at 49.6% [20]. These findings highlight the impact of changes in Asian diets due to globalisation, urbanisation, and Western cultural influences, leading to increased ‘snackification’ and consumption of highly processed foods [53]. Such changes have potentially undermined the assumption of lower acrylamide exposure in Asian diets compared to Western diets. Our study indicates that the rising consumption of snack foods with high acrylamide levels outside main meals can be a significant contributor to acrylamide exposure.

### 3.4. Risk Assessment of Acylamide in Foods Consumed in Singapore

In 2011, the Joint FAO/WHO Expert Committee on Food Additives (JECFA) concluded that the lowest BMDL_10_ of 1.8 × 10^2^ µg/kg bw/day for tumours in the Harderian gland of male mice and 3.1 × 10^2^ µg/kg bw/day for mammary tumours in female rats as reference points for calculation of MOE values [10,54]. In 2015, the EFSA Scientific Panel on Contaminants in the Food Chain (CONTAM Panel) considered the lowest BMDL_10_ of 1.7 × 10^2^ µg/kg bw/day for incidences of Harderian gland adenomas and adenocarcinomas in male B6C3F_1_ mice as reference points for calculation of MOE values [13,55]. In this study, MOE values were calculated for both mean and high consumers using neoplastic-effect-related reference points considered by JECFA and EFSA (Table 6). The calculated MOE values using Equation (2) for both mean and high consumers in Singapore were all less than 10,000, thus indicating potential human health concern to neoplastic effects of acrylamide.

### 3.5. Potential Mitigation Measures to Reduce Dietary Acrylamide Exposure

Our study showed that snack foods consumed outside of main meals are significant contributors to total acrylamide exposure for Singapore consumers, equivalent to main meals. These findings allow for targeted mitigation measures to control and reduce acrylamide occurrence in food. Consumers can moderate their snacking frequency and be mindful of choices, reducing consumption of foods known to have high acrylamide levels (such as French fries, potato chips, crackers, biscuits, and wafers) [56,57]. Choosing fresh and wholesome raw materials over highly processed types during home cooking can also help. Revisiting traditional Asian cooking methods like steaming and boiling for starch-rich vegetables, such as roots and tubes, can further reduce acrylamide formation. The food industry should be encouraged to implement a code of practices or adopt industry guidance from international organisations and national authorities (e.g., Codex Code of Practice for Reduction of Acrylamide in Foods, FoodDrinkEurope ‘Acrylamide ToolBox’) to minimise acrylamide formation in foods [12,14]. Further research is needed to innovate solutions on crop variety selection and farming practices to reduce the natural occurrence of acrylamide precursors (e.g., asparagine) in raw food materials, as well as on food processing techniques to reduce the formation of acrylamide in the final products.

## 4. Conclusions

In this study, we found that foods consumed within and outside the three main meals contributed equally to the total dietary exposure of acrylamide in Singapore for both general and high consumers (95th percentile). Snack foods consumed outside the main meals accounted for 57.4% (general consumer) and 54.6% (high consumer) of the total acrylamide exposure. The estimated mean dietary exposure for the Singapore population was 0.165 µg/kg bw/day for general consumers and 0.392 µg/kg bw/day for high consumers. The calculated MOEs based on reference points considered by JECFA and EFSA were well below 10,000, indicating potential human health concerns related to acrylamide exposure through food consumption. The major contributors to the total dietary exposure of acrylamide were ready-to-eat savouries, vegetables, and composite foods for both general and high consumers. The acrylamide levels were highest in snack foods consumed outside of main meals, particularly in potato-based crackers and chips. On the other hand, foods consumed within main meals showed lower acrylamide levels, with certain starch-rich vegetables (pea, pea sprout, beansprout, long bean, lotus root, potato, and ginger) found with higher concentrations when stir-fried, roasted, deep-fried, or baked.

Food safety is a shared responsibility, with both the food industry and consumers playing vital roles in reducing dietary acrylamide exposure due to the growing ‘snackification’ trend in Singaporean diets. This study emphasised the need for the dissemination of knowledge to advise consumers on practical ways to reduce acrylamide exposure and calls for the adoption of a good code of practices in food production and processing to control acrylamide formation. Furthermore, this study highlights the importance of academic research in finding innovative solutions to control acrylamide formation across the food supply chain, from farm to fork.

## Figures and Tables

**Table 1:** Cooking methods and temperatures used for main meals.

Cooking Methods ^a^	Temperature (°C)
Boil, braise, brew, half-boil, soup, steam, stew	100
Stir fry, pan fry	150
Deep fry	180
Bake	180–200
Roast	200–250

^a^ The cooking duration varied according to the type of food products and cooking methods.

**Table 2:** The MS detector run setting in acrylamide analysis.

Analyte	Retention Time *t*_R_ (min) ^a^	Parent Ion (*m/z*)	Product Ion (*m/z*)	Collision Energy (CE)
Acrylamide	3.61 ± 0.36	72	55	15
Acrylamide-2,3,3-d3	3.57 ± 0.36	75	58	15

^a^ Retention time indicated for the quantitative column, Hypercarb^TM^ Porous Graphitic Carbon HPLC. (±): Uncertainty with potential to result in a shift in retention time for different batches, which is contingent upon the conditions of the column and mobile phase.

**Table 3:** Summary of acrylamide data in 13 food categories commonly consumed as main meals.

Entry	Food Category	Number of Food Products	*n ^a^*	Cooking Methods	LC *^b^* (%)	Range (µg/kg)	Mean (µg/kg)	Median (µg/kg)	SD Mean *^c^*	CV (%)
1	Vegetables	64	120	see entries 1.1 to 1.7	54.0	0.5 *^f^*–478.4	32.2	0.5	7.6	257.9
1.1	Brassica vegetables	4	7	Boil, stir fry *^g^*	28.6	0.5 *^f^*–57.3	21.0	8.9	8.9	112.1
1.2	Fruiting vegetables	8	16	Boil, steam, stir fry *^g^*, RTE *^d^*	75.0	0.5 *^f^*–69.0	7.3	0.5 *^f^*	4.4	241.7
1.3	Leafy vegetables, herbs	17	33	Boil, stir fry *^g^*, RTE *^d^*, soup	54.5	0.5 *^f^*–38.9	6.6	0.5 *^f^*	1.7	148.1
1.4	Legumes	10	16	Boil, stir fry *^g^*, NC *^e^*, pan fry, soup	68.8	0.5 *^f^*–362.9	68.6	0.5 *^f^*	33.2	193.8
1.5	Stalk, stem and bulb vegetables	11	20	Boil, stir fry *^g^*, stew, RTE *^d^*, steam, soup	50.0	0.5 *^f^*–159.0	15.4	1.5	8.1	235.1
1.6	Root and tubers	6	18	Boil, stir fry *^g^*, RTE *^d^*, soup, bake, deep fry, roast *^g^*, steam	38.9	0.5 *^f^*–478.4	101.1	12.2	34.7	145.4
1.7	Fungi, seaweed	7	10	Boil, NC *^e^*, stir fry, soup *^g^*	50.0	0.5 *^f^*–66.4	15.9	2.1	8.5	168.0
2	Vegetable protein	3	6	Braised, stir fry, deep fry *^g^*	83.3	0 *^f^*–3.5	0.6	0 *^f^*	0.6	244.9
3	Fruit and fruit products	34	43	NC *^e,g^*	95.3	0 *^f^*–3.5	0.2	0 *^f^*	0.1	458.6
4	Grain and grain-based products	21	26	Boil, Soup, RTE *^d,g^*, stir fry, NC *^e^*, steam	65.4	0 *^f^*–82.8	8.9	0 *^f^*	3.8	219.3
5	Meat and meat products	17	45	Boil, grill, pan fry, stir fry, stew, roast, steam, soup (liquid, solids), RTE, bake, deep fry *^g^*, braised, NC *^e^*	57.8	0.5 *^f^*–20.1	3.3	0.5 *^f^*	0.7	137.2
6	Eggs and egg products	4	11	Boil, RTE *^d,g^*, NC *^e^*, braised, half-boil, pan fry, steam	90.9	0 *^f^*–4.6	0.4	0 *^f^*	0.4	331.7
7	Milk and dairy products	15	15	NC *^e,g^*, reconstitute	93.3	0 *^f^*–10.4	0.7	0 *^f^*	0.7	387.3
8	Fish and seafood	32	62	Deep fry, pan fry, soup, stir fry, stew *^g^*, NC *^e^*, RTE *^d^*, bake, steam *^g^*, boil, braised	83.9	0 *^f^*–25.4	1.7	0 *^f^*	0.7	318.4
9	Fats and oils	2	4	Stir fry, deep fry, NC *^e^*	100.0	0 *^f^*	0 *^f^*	0 *^f^*	0	-
10	Sauces and condiments	22	25	Boil, NC *^e,g^*, RTE *^d^*, stir fry	44.0	0.5 *^f^*–403.8	38.8	5.5	19.5	250.5
11	Beverages (excluding coffee and coffee substitutes)	10	10	RTE *^d^*, NC *^e^*, brew *^g^*	80.0	0 *^f^*–7.9	1.2	0 *^f^*	0.8	228.5
12	Bakery products (excluding biscuits and wafers)	6	6	NC *^e,g^*	16.7	0.5 *^f^*–14.4	9.9	10.9	2.0	48.7
13	Composite foods	12	12	Pan fry, boil *^g^*, deep fry, steam, NC *^e^*, RTE *^d^*	50.0	0.5 *^f^*–57.6	17.7	2.2	7.1	138.4

*^a^ n*: number of samples. *^b^* LC: percentage of left-censored results. *^c^* SD mean: Standard deviation of the mean. *^d^* RTE: Ready-to-eat. *^e^* NC: No cooking. *^f^* Acrylamide concentrations less than LOD were considered as 0 or 1/2 LOD depending on the LC (%) in each food category. *^g^* This cooking method produced the highest acrylamide concentrations in each food category.

**Table 4:** Summary of acrylamide data in snack food products commonly consumed outside the main meals.

Entry	Food Category (Food Products)	*n ^a^*	LC *^b^* (%)	Range (µg/kg)	Mean (µg/kg)	Median (µg/kg)	SD Mean *^c^*	CV (%)
1	RTE Savouries	295	21.4	0.5 *^d^*–2866.5	198.0	74.2	19.7	171.0
1.1	Crackers and chips	149	22.1	0.5 *^d^*–2866.5	248.6	54.7	36.5	179.0
	Potato-based	36	0	81.8–2095.8	732.7	650.8	85.2	69.8
	Fruits (banana, belinjau, jackfruit)	10	30.0	0.5 *^d^*–2866.5	339.7	72.9	281.2	261.7
	Wheat flour	18	22.2	0.5 *^d^*–600.6	131.4	47.9	42.0	135.5
	Rice flour, dhall flour	22	4.5	0.5 *^d^*–458.4	113.8	43.3	29.4	121.4
	Vegetables (cassava, corn, popcorn, sorghum, tapioca, taro, tortilla)	41	31.7	0.5 *^d^*–238.8	48.8	30.1	8.9	117.0
	Seafood (fish, fish skin, scallops, shrimp, prawns)	22	54.5	0.5 *^d^*–132.7	18.4	0.5 *^d^*	7.2	183.7
1.2	Biscuits and wafers	98	8.2	0.5 *^d^*–673.7	195.7	155.2	16.4	83.0
1.3	Cereal-based snack bars and popcorns	14	7.1	0.5 *^d^*–375.3	126.9	74.5	32.2	94.9
1.4	Dried snack food (cuttlefish, fish, squid titbits)	34	61.8	0.5 *^d^*–93.4	11.8	0.5 *^d^*	3.5	170.5
2	Beverages (Coffee) *^e^*	51	0	6–593.3 (0.5–30.0)	108.4 (8.7)	84.0 (6.1)	12.9 (0.9)	85.2 (73.0)
	Roast coffee (dry) *^e^*	25	0	45.8–593.3 (2.6–19.3)	146.0 (7.4)	109.0 (6.1)	22.2 (0.8)	75.9 (53.6)
	Instant coffee (dry)* ^e^*	6	0	25.7–102.1 (3.4–14.3)	60.4 (7.3)	57.4 (5.5)	10.6 (1.7)	42.8 (55.6)
	Coffee substitute (dry) exclusively from cereals *^e^*	20	0	6.0–212.0 (0.5–30.0)	75.8 (10.8)	75.6 (9.6)	12.4 (1.9)	73.4 (80.3)
3	Nuts	40	35.0	0.5 *^d^*–247.2	41.4	34.4	8.1	123.9
	Almond	4	50.0	0.5 *^d^*–71.3	33.9	31.8	19.3	114.2
	Brazil nut kernels	1	-	0.5 *^d^*	0.5 *^d^*	0.5 *^d^*	-	-
	Cashew	7	28.6	0.5 *^d^*–72.0	22.7	11.4	10.4	121.1
	Chestnuts	2	0	14.0–77.9	46.0	46.0	32.0	98.3
	Peanuts	13	46.2	0.5 *^d^*–188.5	36.8	32.8	14.6	142.9
	Pistachio	1	-	0.5 *^d^*	0.5 *^d^*	0.5 *^d^*	-	-
	Walnut	2	50.0	0.5 *^d^*–38.8	19.7	19.7	19.2	137.8
	Mixed nuts	10	10.0	0.5 *^d^*–247.2	75.3	57.4	20.8	87.5

*^a^ n*: number of samples. *^b^* LC: percentage of left-censored results. *^c^* SD mean: Standard deviation of the mean. *^d^* Acrylamide concentrations less than LOD were considered as 1/2 LOD. *^e^* The results for coffee beverages were analysed in powder equivalent; the results expressed in the parenthesis took into account the dilution factor calculated from the manufacturer’s recommended amount of boiled water (volume) to add into each serving (grams) as indicated on the product labelling. When there is no such information indicated, the dilution factor of 18 was considered [38].

**Table 5:** Summary of dietary exposures of the population in Singapore to acrylamide in food category/products.

		Mean Consumer		High Consumer (95th Percentile)	
Entry	Food Category/Products	Dietary Exposure of Acrylamide (µg/kg bw/Day)	Percentage Contribution (%)	Dietary Exposure of Acrylamide (µg/kg bw/Day)	Percentage Contribution (%)
1	Vegetable	2.1 × 10^−2^	12.9	6.5 × 10^−2^	16.7
2	Vegetable protein	4.3 × 10^−4^	0.3	9.7 × 10^−4^	0.2
3	Fruit and fruit products	9.1 × 10^−5^	0.1	1.7 × 10^−4^	<0.1
4	Grains and grain-based products	9.5 × 10^−3^	5.8	1.6 × 10^−2^	4.0
5	Meat and meat products	2.3 × 10^−3^	1.4	5.4 × 10^−3^	1.4
6	Eggs and egg products	1.3 × 10^−4^	0.1	3.5 × 10^−4^	0.1
7	Milk and dairy products	9.1 × 10^−5^	0.1	2.4 × 10^−4^	0.1
8	Fish and seafood	5.9 × 10^−4^	0.4	2.0 × 10^−3^	0.5
9	Fats and oils	0	0	0	0
10	Sauces and condiments	7.5 × 10^−3^	4.6	2.4 × 10^−2^	6.1
11	Beverages (excluding coffee and coffee substitutes)	2.9 × 10^−3^	1.8	6.3 × 10^−3^	1.6
12	Bakery products (excluding biscuits and wafers)	5.8 × 10^−3^	3.5	1.2 × 10^−2^	3.0
13	Composite foods	2.0 × 10^−2^	11.9	4.5 × 10^−2^	11.5
Dietary exposure (within 3 main meals) *^a^*		0.070	42.6	0.178	45.4
14	RTE savouries	7.4 × 10^−2^	44.8	1.6 × 10^−1^	40.4
15	Beverages (Coffee)	1.3 × 10^−2^	8.1	2.8 × 10^−2^	7.0
16	Nuts	7.4 × 10^−3^	4.5	2.8 × 10^−2^	7.2
Dietary exposure (outside main meals) *^b^*		0.095	57.4	0.214	54.6
Total Dietary Exposure (within and outside main meals) *^c^*		0.165	100	0.392	100

*^a^* Dietary exposure (within 3 main meals) was calculated from the summation of entries 1–13. *^b^* Dietary exposure (outside main meals) was calculated from the summation of entries 14–16. *^c^* Total dietary exposure (within and outside main meals) was calculated from the summation of entries 1–16.

**Table 6:** The calculated MOE values in this study using the reference points considered by JECFA and EFSA.

Reference Point (µg/kg bw/Day)	Critical Effect	Calculated MOE in This Study *^c^*
		General (High) Consumers
1.8 × 10^2^ (BMDL_10_) *^a^*	Tumours in the Harderian gland of male mice *^a^*	1092 (459)
3.1 × 10^2^ (BMDL_10_) *^a^*	Mammary tumours in female rats *^a^*	1880 (790)
1.7 × 10^2^ (BMDL_10_) *^b^*	Neoplastic effects in mice *^b^*	1031 (433)

*^a^* JECFA (2011) considered tumours in the Harderian gland of male mice and mammary tumours in female rats as appropriate reference points (points of departure) based on Beland’s (2010) study. *^b^* EFSA (2015) considered incidences of Harderian glands in male mice as appropriate reference points (points of departure) based on NTP (2012) study. *^c^* The MOE values were calculated using Equation (2) with the estimated dietary exposure to acrylamide in this study and the reference points used by JECFA (2011) and EFSA (2015).

## Data Availability

Data are contained within the article and Supplementary Materials.

## Supplementary Materials

The following supporting information can be downloaded at: https://www.mdpi.com/article/10.3390/foods12163022/s1, Table S1: Summary of results for vegetable category (brassica vegetables); Table S2: Summary of results for vegetable category (fruiting vegetables); Table S3: Summary of results for vegetable category (leafy vegetables, herbs); Table S4: Summary of results for vegetable category (legumes); Table S5: Summary of results for vegetable category (stalk, stem and bulb vegetables); Table S6: Summary of results for vegetable category (root and tubers); Table S7: Summary of results for vegetable category (fungi, seaweed); Table S8: Summary of results for vegetable protein category; Table S9: Summary of results for fruits and fruit products category; Table S10: Summary of results for grain and grain-based products category; Table S11: Summary of results for meat and meat products category; Table S12: Summary of results for eggs and egg products category; Table S13: Summary of results for milk and dairy products category; Table S14: Summary of results for fish and seafood category; Table S15: Summary of results for fats and oils category; Table S16: Summary of results for sauces and condiments; Table S17: Summary of results for beverages category (excluding coffee and coffee substitutes); Table S18: Summary of results for bakery products category (excluding biscuits and wafers); Table S19: Summary of results for composite foods category; Table S20: Summary of results for RTE savouries category (crackers and chips, biscuits and wafers, cereal-based snack bars, dried snack food); Table S21: Summary of results for beverages category (coffee and coffee substitutes); Table S22: Summary of results for nuts. Figure S1: Contribution of individual food category from within and outside of main meals to the total dietary exposure of acrylamide for general consumers; Figure S2: Contribution of individual food category from within and outside of main meals to the total dietary exposure of acrylamide for high consumers.
