# Supplementary material for: Occurrence and Dietary Exposure to Acrylamide from Foods Consumed within and outside Main Meals in Singapore
Source: Foods. 2023 Aug 11;12(16):3022. doi: 10.3390/foods12163022 (PMC10453405; doi:10.3390/foods12163022)
Supplement: Supplementary file 1 [file foods-12-03022-s001.zip › foods-2533175-supplementary.pdf]

Supporting Information

Occurrence and Dietary Exposure to Acrylamide form Foods Consumed Within and Outside Main Meals in Singapore

Wesley Zongrong Yu [1], Ping Shen [1,*], Ignatius Lim [1], Raymond Rong Sheng Shi [1], Miaohua Cai [1], Yee Soon Chin [1], Ai Jin Tay [1], Wei Min Ang [1], Jun Cheng Er [1], Geraldine Songlen Lim [1], Yuansheng Wu [1], Angela Li [1], Kyaw Thu Aung [1,2], and Sheot Harn Chan [1,3]

[1] National Centre for Food Science, Singapore Food Agency, 7 International Business Park, Singapore 609919, Singapore. [2] School of Biological Sciences, Nanyang Technological University, 60 Nanyang Drive, Singapore 637551, Singapore. [3] Department of Food Science and Technology, 2 Science Drive 2, Faculty of Science, National University of Singapore, Singapore 117543, Singapore.

** Correspondence: ping_shen@sfa.gov.sg*

TABLE OF CONTENTS

Food commonly consumed within the 3 main meals tested for the occurrence of acrylamide: **Table S1 to S19**	……………………………**S-2 – S-33**
Food commonly consumed between the main meals tested for the occurrence of acrylamide: **Table S20 to S22**	………………………….**S-34 – S-62**
Contribution of individual food category from within and outside of main meals to the overall dietary exposure of acrylamide for consumers in Singapore: **Figure S1 and S2**	………………………….**S-63 – S-64**

Foods commonly consumed within the 3 main meals tested for the occurrence of acrylamide

Table S1 to S19 were foods selected on the basis that they are commonly consumed within the main meals in Singapore. There are a total of 13 food categories with 261 food products. By taking into account the different cooking methods, there were a total of 385 food samples tested for their acrylamide concentration (μg/kg or ppb) using LC-MS/MS with a limit of detection (LOD) of 1.0 μg/kg. Those samples not detected with acrylamide (i.e. concentration below the LOD) are treated according to the WHO recommendation on the evaluation of low-level contaminant of food. The concentrations below LOD were set at 0 when the food category contains more than 60% of samples not detected with acrylamide; while the concentration below LOD were set at half the LOD when the food category contained less than 60% of samples not detected with acrylamide. The food consumption data of Singapore population were obtained from 24-hour dietary recall surveys for consumers from age 15 to 92 years old. The consumption amount (g/kg body weight or bw) of mean and high consumers (P95) were determined. The dietary exposure of acrylamide from each food product was calculated by multiplying the concentration (μg/kg) and consumption amount (g/kg bw) together with a unit conversion factor (10^{-3}).

Table S1: Summary of results for vegetables category (brassica vegetables) (n=7)

	**Samples and Preparation**		**LC-MS/MS Analysis of Acrylamide**	**24-hour Dietary Recall Survey on Consumers (eaters only) from aged 15 to 92 years old**					
No.	Food Product	Cooking Method	Concentration (μg/kg)	Number of consumers surveyed	Mean Body Weight, (kg bw)	Consumption of mean consumers (g/kg bw)	Consumption of high consumers (P95) (g/kg bw)	Dietary exposure of mean consumers (μg/kg bw/day)	Dietary exposure of high consumers (P95) (μg/kg bw/day)
1	Cabbage	Boil	0.5	176	66.0	0.523	1.412	0.0003	0.0007

2	Cabbage	Stir Fry	8.9	176	66.0	0.523	1.412	0.0047	0.0126
3	Broccoli	Boil	1.8	248	64.6	0.537	1.578	0.0010	0.0028
4	Broccoli	Stir Fry	57.3	248	64.6	0.537	1.578	0.0308	0.0904
5	Cauliflower	Stir Fry	37.7	86	64.9	0.929	1.745	0.0350	0.0658
6	Cauliflower	Boil	40.4	86	64.9	0.929	1.745	0.0376	0.0705
7	Preserved / pickled vegetable	Boil	0.5	39	62.3	0.504	1.818	0.0003	0.0009

Table S2: Summary of results for vegetables category (fruiting vegetables) (n=16)

	**Samples and Preparation**		**LC-MS/MS Analysis of Acrylamide**	**24-hour Dietary Recall Survey on Consumers (eaters only) from aged 15 to 92 years old**					
No.	Food Product	Cooking Method	Concentration (µg/kg)	Number of consumers surveyed	Mean Body Weight, (kg bw)	Consumption of mean consumers (g/kg bw)	Consumption of high consumers (P95) (g/kg bw)	Dietary exposure of mean consumers (µg/kg bw/day)	Dietary exposure of high consumers (P95) (µg/kg bw/day)
1	Brinjal	Boil	0.5	180	63.3	0.322	0.957	0.0002	0.0005
2	Brinjal	Steam	0.5	180	63.3	0.322	0.957	0.0002	0.0005
3	Brinjal	Stir Fry	13.0	180	63.3	0.322	0.957	0.0042	0.0124
4	Capsicum	Stir Fry	6.2	86	66.1	0.119	0.526	0.0007	0.0033

5	Capsicum	Boil	0.5	86	66.1	0.119	0.526	0.0001	0.0003
6	Chilli	Boil	0.5	105	64.7	0.166	0.521	0.0001	0.0003
7	Chilli	RTE	0.5	105	64.7	0.166	0.521	0.0001	0.0003
8	Chilli	Stir Fry	69.0	105	64.7	0.166	0.521	0.0115	0.0360
9	Bitter gourd	Boil	0.5	151	64.0	0.646	2.485	0.0003	0.0012
10	Bitter gourd	Stir Fry	0.5	151	64.0	0.646	2.485	0.0003	0.0012
11	Bottle gourd	Stir Fry	0.5	6	57.3	5.430	8.523	0.0027	0.0043
12	Winter melon	Boil	0.5	18	66.2	1.889	5.521	0.0009	0.0028
13	Tomato	Boil	0.5	491	65.5	1.002	5.270	0.0005	0.0026
14	Tomato	RTE	0.5	491	65.5	1.002	5.270	0.0005	0.0026
15	Tomato	Stir Fry	0.5	491	65.5	1.002	5.270	0.0005	0.0026
16	Zucchini	Stir Fry	21.8	4	65.0	0.752	1.206	0.0164	0.0263

Table S3: Summary of results for vegetables category (leafy vegetables, herbs) (n=33)

	**Samples and Preparation**		**LC-MS/MS Analysis of Acrylamide**	**24-hour Dietary Recall Survey on Consumers (eaters only) from aged 15 to 92 years old**					
No.	Food Product	Cooking Method	Concentration (µg/kg)	Number of consumers surveyed	Mean Body Weight, (kg bw)	Consumption of mean consumers (g/kg bw)	Consumption of high consumers	Dietary exposure of mean consumers	Dietary exposure of high consumers

							(P95) (g/kg bw)	(µg/kg bw/day)	(P95) (µg/kg bw/day)
1	Bai cai	Boil	0.5	227	65.8	0.805	2.768	0.0004	0.0014
2	Bai cai	Stir Fry	12.0	227	65.8	0.805	2.768	0.0097	0.0332
3	Xiao bai cai	Boil	0.5	227	65.8	0.805	2.768	0.0004	0.0014
4	Xiao bai cai	Stir Fry	4.2	227	65.8	0.805	2.768	0.0034	0.0116
5	Basil	Stir Fry	38.9	16	63.5	0.250	1.315	0.0097	0.0512
6	Bayam	Boil	0.5	5	60.0	0.871	1.337	0.0004	0.0007
7	Bayam	Stir Fry	0.5	5	60.0	0.871	1.337	0.0004	0.0007
8	Chye sim	Stir Fry	20.7	478	65.7	0.732	2.732	0.0152	0.0565
9	Chye sim	Boil	0.5	478	65.7	0.732	2.732	0.0004	0.0014
10	Coriander	RTE	2.8	540	66.2	0.038	0.140	0.0001	0.0004
11	Coriander	Boil	0.5	540	66.2	0.038	0.140	0.0000	0.0001
12	Coriander	Stir Fry	32.2	540	66.2	0.038	0.140	0.0012	0.0045
13	Kale	Stir Fry	4.4	9	61.3	0.490	0.906	0.0022	0.0040
14	Kale	Boil	4.6	9	61.3	0.490	0.906	0.0023	0.0042
15	Kailan	Boil	0.5	120	66.2	0.784	2.407	0.0004	0.0012
16	Kailan	Stir Fry	8.2	120	66.2	0.784	2.407	0.0064	0.0197
17	Kang kong	Boil	0.5	136	64.3	0.341	1.434	0.0002	0.0007
18	Kang kong	Stir Fry	18.5	136	64.3	0.341	1.434	0.0063	0.0265

19	Mint leaf	RTE	7.1	6	57.0	0.147	0.359	0.0010	0.0025
20	Pandan leaf	Soup	0.5	417	66.8	0.022	0.043	0.0000	0.0000
21	Pandan leaf	Soup	0.5	417	66.8	0.022	0.043	0.0000	0.0000
22	Sweet potato leaf	Boil	0.5	16	58.8	1.743	2.640	0.0009	0.0013
23	Sweet potato leaf	Stir Fry	8.3	16	58.8	1.743	2.640	0.0145	0.0219
24	Lettuce	Stir Fry	12.7	253	64.3	0.326	0.775	0.0041	0.0098
25	Lettuce	Boil	0.5	253	64.3	0.326	0.775	0.0002	0.0004
26	Lettuce	RTE	0.5	253	64.3	0.326	0.775	0.0002	0.0004
27	Nai bai	Boil	0.5	11	70.5	0.412	0.912	0.0002	0.0005
28	Nai bai	Stir Fry	22.2	11	70.5	0.412	0.912	0.0091	0.0202
29	Spinach	Stir Fry	13.6	185	64.1	0.961	2.826	0.0131	0.0384
30	Spinach	Boil	0.5	185	64.1	0.961	2.826	0.0005	0.0014
31	Malabar	Stir Fry	0.5	4	69.0	0.292	0.459	0.0001	0.0002
32	Malabar	Boil	0.5	4	69.0	0.292	0.459	0.0001	0.0002
33	Watercress	Boil	0.5	19	65.6	1.338	2.963	0.0007	0.0015

Table S4: Summary of results for vegetables category (legumes) (n=16)

	Samples and Preparation	LC-MS/MS Analysis of Acrylamide	24-hour Dietary Recall Survey on Consumers (eaters only) from aged 15 to 92 years old

No.	Food Product	Cooking Method	Concentration (µg/kg)	Number of consumers surveyed	Mean Body Weight, (kg bw)	Consumption of mean consumers (g/kg bw)	Consumption of high consumers (P95) (g/kg bw)	Dietary exposure of mean consumers (µg/kg bw/day)	Dietary exposure of high consumers (P95) (µg/kg bw/day)
1	Long bean	Stir Fry	93.9	149	64.1	0.413	1.058	0.0388	0.0994
2	Dried bean	Boil	0.5	6	68.3	0.476	1.260	0.0002	0.0006
3	Beansprout	Boil	0.5	512	66.6	0.368	1.829	0.0002	0.0009
4	Beansprout	Stir Fry	324.4	512	66.6	0.368	1.829	0.1195	0.5933
5	Lentil	Stir Fry	3.3	19	70.1	1.674	3.888	0.0055	0.0128
6	Lentil	Boil	0.5	19	70.1	1.674	3.888	0.0008	0.0019
7	Pea	Boil	0.5	56	61.8	0.373	1.279	0.0002	0.0006
8	Soy beancurd tofu tau kwa taupok tempeh	Pan fry	0.5	73	66.2	0.362	0.783	0.0002	0.0004
9	Soy beancurd tofu tau kwa taupok tempeh	Boil	0.5	129	66.9	0.536	1.822	0.0003	0.0009
10	Pea	Stir Fry	362.9	56	61.8	0.373	1.279	0.1354	0.4640
11	Pea sprout	Stir Fry	307.4	7	62.9	0.374	0.791	0.1151	0.2430
12	Pea sprout	Boil	0.5	7	62.9	0.374	0.791	0.0002	0.0004
13	Soy milk	No cooking	0.5	68	64.6	2.416	4.690	0.0012	0.0023

14	Fermented soybean product	Stir Fry	0.5	1	95.0	0.084	0.084	0.0000	0.0000
15	Soybean	Soup	0.5	70	62.6	0.393	0.834	0.0002	0.0004
16	Soybean	Soup	0.5	70	62.6	0.393	0.834	0.0002	0.0004

Table S5: Summary of results for vegetables category (stalk, stem and bulb vegetables) (n=20)

	**Samples and Preparation**		**LC-MS/MS Analysis of Acrylamide**	**24-hour Dietary Recall Survey on Consumers (eaters only) from aged 15 to 92 years old**					
No.	Food Product	Cooking Method	Concentration (µg/kg)	Number of consumers surveyed	Mean Body Weight, (kg bw)	Consumption of mean consumers (g/kg bw)	Consumption of high consumers (P95) (g/kg bw)	Dietary exposure of mean consumers (µg/kg bw/day)	Dietary exposure of high consumers (P95) (µg/kg bw/day)
1	Asparagus	Boil	0.5	21	64.1	0.750	3.150	0.0004	0.0016
2	Bamboo shoot	Boil	0.5	12	64.8	0.492	1.416	0.0002	0.0007
3	Chinese celery	Stir Fry	10.8	67	67.1	0.269	1.048	0.0029	0.0113
4	Chinese celery	Boil	0.5	67	67.1	0.269	1.048	0.0001	0.0005
5	Celery	Boil	0.5	67	67.1	0.269	1.048	0.0001	0.0005
6	Celery	Stir Fry	27.3	67	67.1	0.269	1.048	0.0074	0.0286
7	Garlic	Stew	26.4	1417	65.5	0.096	0.258	0.0025	0.0068

8	Garlic	Stir Fry	51.5	4	57.5	0.046	0.048	0.0024	0.0025
9	Garlic	Soup	2.6	1417	65.5	0.096	0.258	0.0002	0.0007
10	Garlic	Soup	0.5	1417	65.5	0.096	0.258	0.0000	0.0001
11	Ginger	Soup	0.5	1052	65.7	0.089	0.215	0.0000	0.0001
12	Ginger	Soup	0.5	1052	65.7	0.089	0.215	0.0000	0.0001
13	Ginger	Stir Fry	159.0	1052	65.7	0.089	0.215	0.0142	0.0341
14	Ginger	Steam	4.9	1052	65.7	0.089	0.215	0.0004	0.0011
15	Dried lily bulb	Boil	8.0	1	50.0	0.065	0.065	0.0005	0.0005
16	Lily bulb	Stir Fry	0.5	2	52.5	0.176	0.248	0.0001	0.0001
17	Onion	Boil	0.5	452	65.7	0.454	1.565	0.0002	0.0008
18	Onion	Stir Fry	4.0	452	65.7	0.454	1.565	0.0018	0.0062
19	Spring onion	RTE	0.5	1007	65.5	0.059	0.135	0.0000	0.0001
20	Turmeric	Boil	8.8	183	67.4	0.045	0.200	0.0004	0.0018

Table S6: Summary of results for vegetables category (root and tubers) (n=18)

	Samples and Preparation	LC-MS/MS Analysis of Acrylamide	24-hour Dietary Recall Survey on Consumers (eaters only) from aged 15 to 92 years old

No.	Food Product	Cooking Method	Concentration (µg/kg)	Number of consumers surveyed	Mean Body Weight, (kg bw)	Consumption of mean consumers (g/kg bw)	Consumption of high consumers (P95) (g/kg bw)	Dietary exposure of mean consumers (µg/kg bw/day)	Dietary exposure of high consumers (P95) (µg/kg bw/day)
1	Carrot	Stir Fry	59.6	431	65.0	0.669	2.284	0.0399	0.1361
2	Carrot	RTE	5.4	431	65.0	0.669	2.284	0.0036	0.0123
3	Carrot	Boil	4.1	431	65.0	0.669	2.284	0.0027	0.0094
4	Lotus root	Soup	0.5	47	61.3	0.608	2.095	0.0003	0.0010
5	Lotus root	Soup	0.5	47	61.3	0.608	2.095	0.0003	0.0010
6	Lotus root	Stir Fry	213.7	47	61.3	0.608	2.095	0.1299	0.4478
7	Potato	Boil	18.9	367	65.5	1.099	3.658	0.0208	0.0691
8	Potato	Bake	185.9	367	65.5	1.099	3.658	0.2043	0.6801
9	Potato	Stir Fry	188.6	367	65.5	1.099	3.658	0.2073	0.6900
10	Potato	Deep fry	244.3	367	65.5	1.099	3.658	0.2685	0.8938
11	Potato	Roast	478.4	367	65.5	1.099	3.658	0.5258	1.7502
12	Pumpkin	Boil	0.5	38	60.1	1.809	7.041	0.0009	0.0035
13	Sweet potato	Boil	0.5	73	64.8	0.646	1.382	0.0003	0.0007
14	Sweet potato	Roast	376.6	73	64.8	0.646	1.382	0.2432	0.5203
15	Sweet potato	Bake	41.6	73	64.8	0.646	1.382	0.0268	0.0574

16	Sweet potato	Steam	0.5	73	64.8	0.646	1.382	0.0003	0.0007
17	Yam	Steam	0.5	45	63.0	0.967	3.597	0.0005	0.0018
18	Yam	Boil	0.5	45	63.0	0.967	3.597	0.0005	0.0018

Table S7: Summary of results for vegetables category (fungi, seaweed) (n=10)

	**Samples and Preparation**		**LC-MS/MS Analysis of Acrylamide**	**24-hour Dietary Recall Survey on Consumers (eaters only) from aged 15 to 92 years old**					
No.	Food Product	Cooking Method	Concentration (μg/kg)	Number of consumers surveyed	Mean Body Weight, (kg bw)	Consumption of mean consumers (g/kg bw)	Consumption of high consumers (P95) (g/kg bw)	Dietary exposure of mean consumers (μg/kg bw/day)	Dietary exposure of high consumers (P95) (μg/kg bw/day)
1	Black jelly fungus	Boil	0.5	46	64.7	0.306	0.822	0.0002	0.0004
2	Canned mushroom	Boil	3.6	1	48.0	0.318	0.318	0.0011	0.0011
3	Dried mushroom	Boil	0.5	261	64.4	0.035	0.098	0.0000	0.0000
4	Dried seaweed	No cooking	65.3	44	58.4	0.046	0.120	0.0030	0.0078
5	Fresh mushroom	Boil	4.7	115	63.8	0.209	0.493	0.0010	0.0023
6	Fresh mushroom	Stir Fry	0.5	115	63.8	0.209	0.493	0.0001	0.0002
7	Seaweed	Soup	66.4	3	57.7	2.009	4.440	0.1334	0.2948
8	Seaweed	Soup	16.9	3	57.7	2.009	4.440	0.0340	0.0750

9	Seaweed	Boil	0.5	3	57.7	2.009	4.440	0.0010	0.0022
10	White jelly fungus	Boil	0.5	13	66.8	0.379	1.306	0.0002	0.0007

Table S8: Summary of results for vegetable protein category (n=6)

	**Samples and Preparation**		**LC-MS/MS Analysis of Acrylamide**	**24-hour Dietary Recall Survey on Consumers (eaters only) from aged 15 to 92 years old**					
No.	Food Product	Cooking Method	Concentration (µg/kg)	Number of consumers surveyed	Mean Body Weight, (kg bw)	Consumption of mean consumers (g/kg bw)	Consumption of high consumers (P95) (g/kg bw)	Dietary exposure of mean consumers (µg/kg bw/day)	Dietary exposure of high consumers (P95) (µg/kg bw/day)
1	Mock meat	Braised	0.0	9	61.2	0.733	1.656	0.0000	0.0000
2	Mock meat (gluten based)	Stir Fry	0.0	9	61.2	0.733	1.656	0.0000	0.0000
3	Mock meat (gluten based)	Braised	0.0	9	61.2	0.733	1.656	0.0000	0.0000
4	Mock meat (soy based)	Braised	0.0	9	61.2	0.733	1.656	0.0000	0.0000
5	Mock meat (soy based)	Stir Fry	0.0	9	61.2	0.733	1.656	0.0000	0.0000
6	Mock meat (soy based)	Deep fry	3.5	9	61.2	0.733	1.656	0.0026	0.0058

Table S9: Summary of results for fruits and fruit products category (n=43)

No.	Samples and Preparation		LC-MS/MS Analysis of Acrylamide	24-hour Dietary Recall Survey on Consumers (eaters only) from aged 15 to 92 years old					
	Food Product	Cooking Method	Concentration (µg/kg)	Number of consumers surveyed	Mean Body Weight, (kg bw)	Consumption of mean consumers (g/kg bw)	Consumption of high consumers (P95) (g/kg bw)	Dietary exposure of mean consumers (µg/kg bw/day)	Dietary exposure of high consumers (P95) (µg/kg bw/day)
1	Aloe vera	No cooking	0.0	2	60.0	0.079	0.104	0.0000	0.0000
2	Apple	No cooking	0.0	275	65.4	1.606	3.542	0.0000	0.0000
3	Avocado	No cooking	0.0	47	63.1	1.329	2.781	0.0000	0.0000
4	Avocado	No cooking	0.0	47	63.1	1.329	2.781	0.0000	0.0000
5	Banana	No cooking	0.0	182	67.1	1.517	3.198	0.0000	0.0000
6	Blueberry	No cooking	0.0	47	61.0	2.993	7.449	0.0000	0.0000
7	Coconut flesh	No cooking	0.0	6	63.8	0.236	0.414	0.0000	0.0000
8	COCONUT MILK	No cooking	0.0	145	65.8	0.623	1.578	0.0000	0.0000
9	Coconut water	No cooking	0.0	30	62.3	2.980	6.053	0.0000	0.0000
10	Dragonfruit	No cooking	0.0	35	68.0	0.516	1.259	0.0000	0.0000
11	Dragonfruit	No cooking	0.0	35	68.0	0.516	1.259	0.0000	0.0000
12	Durian	No cooking	3.5	13	67.9	0.967	1.651	0.0034	0.0058

13	Grape	No cooking	0.0	4	62.5	1.547	3.023	0.0000	0.0000
14	Guava	No cooking	0.0	42	65.2	2.008	4.342	0.0000	0.0000
15	Jackfruit	No cooking	0.0	19	67.6	0.618	0.917	0.0000	0.0000
16	Jam	No cooking	0.0	8	61.1	0.162	0.227	0.0000	0.0000
17	Kaya	No cooking	3.2	186	65.5	0.162	0.438	0.0005	0.0014
18	Kiwi	No cooking	0.0	45	64.3	1.205	2.558	0.0000	0.0000
19	Kiwi	No cooking	0.0	45	64.3	1.205	2.558	0.0000	0.0000
20	Lime / calamansi	No cooking	0.0	148	66.7	0.191	0.625	0.0000	0.0000
21	Longan	No cooking	0.0	8	62.5	0.294	0.509	0.0000	0.0000
22	Mandarin orange	No cooking	0.0	12	62.5	1.385	2.261	0.0000	0.0000
23	Mango	No cooking	0.0	34	64.9	1.029	1.935	0.0000	0.0000
24	Mango	No cooking	0.0	34	64.9	1.029	1.935	0.0000	0.0000
25	Mangosteen	No cooking	0.0	2	70.0	2.039	2.172	0.0000	0.0000
26	Orange	No cooking	0.0	126	65.6	1.881	4.285	0.0000	0.0000
27	Orange	No cooking	0.0	126	65.6	1.881	4.285	0.0000	0.0000
28	Papaya	No cooking	0.0	57	62.8	2.434	5.363	0.0000	0.0000
29	Peach	No cooking	0.0	7	56.9	1.081	2.091	0.0000	0.0000
30	Peach	No cooking	0.0	7	56.9	1.081	2.091	0.0000	0.0000
31	Pear	No cooking	0.0	28	67.7	0.999	1.617	0.0000	0.0000
32	Pear	No cooking	0.0	28	67.7	0.999	1.617	0.0000	0.0000

33	Persimmon	No cooking	0.0	11	65.5	0.382	1.282	0.0000	0.0000
34	Persimmon	No cooking	0.0	11	65.5	0.382	1.282	0.0000	0.0000
35	Pineapple	No cooking	0.0	117	65.6	1.040	4.753	0.0000	0.0000
36	Plum	No cooking	0.0	4	56.5	0.207	0.256	0.0000	0.0000
37	Pomelo	No cooking	0.0	6	61.0	1.155	2.147	0.0000	0.0000
38	Pomelo	No cooking	0.0	6	61.0	1.155	2.147	0.0000	0.0000
39	SOURSOP	No cooking	0.0	2	72.0	0.897	1.440	0.0000	0.0000
40	Starfruit	No cooking	0.0	2	60.5	0.687	0.851	0.0000	0.0000
41	Strawberry	No cooking	0.0	49	60.8	0.712	2.343	0.0000	0.0000
42	Water chestnut	No cooking	0.0	48	64.4	0.225	0.812	0.0000	0.0000
43	Watermelon	No cooking	0.0	60	65.2	4.071	9.756	0.0000	0.0000

Table S10: Summary of results for grain and grain-based products category (n=26)

	Samples and Preparation		LC-MS/MS Analysis of Acrylamide	24-hour Dietary Recall Survey on Consumers (eaters only) from aged 15 to 92 years old					
No.	Food Product	Cooking Method	Concentration (µg/kg)	Number of consumers surveyed	Mean Body Weight, (kg bw)	Consumption of mean consumers (g/kg bw)	Consumption of high consumers (P95) (g/kg bw)	Dietary exposure of mean consumers (µg/kg bw/day)	Dietary exposure of high consumers (P95) (µg/kg bw/day)

1	Barley	Soup	0.0	12	59.9	1.267	4.270	0.0000	0.0000
2	Barley	Soup	0.0	12	59.9	1.267	4.270	0.0000	0.0000
3	Breakfast cereal	RTE	82.8	109	61.5	0.330	0.748	0.0274	0.0620
4	Brown rice	Stir Fry	4.2	209	63.3	1.729	4.505	0.0073	0.0189
5	Brown rice	Steam	0.0	209	63.3	1.729	4.505	0.0000	0.0000
6	Buckwheat noodle	Boil	0.0	13	66.8	3.996	7.958	0.0000	0.0000
7	Chapati	No cooking	50.2	33	66.4	0.577	1.211	0.0290	0.0608
8	Coconut rice	Steam	4.5	6	61.5	1.954	3.572	0.0088	0.0161
9	Corn	Boil	0.0	3	63.7	2.193	4.894	0.0000	0.0000
10	GLUTINOUS RICE	Steam	0.0	68	66.2	0.699	3.104	0.0000	0.0000
11	Instant noodle	Boil	7.1	22	62.1	2.736	5.113	0.0194	0.0363
12	Noodles wheat (e.g. ban mian)	Boil	0.0	144	62.2	2.645	5.851	0.0000	0.0000
13	Oat	Boil	0.0	171	62.7	0.803	2.567	0.0000	0.0000
14	Pasta	Boil	0.0	158	66.2	1.311	4.666	0.0000	0.0000
15	Rice dumpling	No cooking	25.8	13	61.1	2.191	3.284	0.0565	0.0847
16	Rice noodle	Boil	0.0	738	65.0	1.638	5.077	0.0000	0.0000
17	Rice noodle	Stir Fry	0.0	738	65.0	1.638	5.077	0.0000	0.0000
18	Roti prata	No cooking	28.5	1	60.0	2.593	2.593	0.0739	0.0739
19	Thosai	No cooking	25.5	67	66.3	0.772	1.959	0.0197	0.0500

20	Udon	Boil	0.0	23	69.2	0.938	1.645	0.0000	0.0000
21	Vermicelli	Boil	0.0	83	65.6	1.354	2.578	0.0000	0.0000
22	White basmati rice	Boil	3.0	139	69.1	1.582	3.390	0.0047	0.0102
23	White basmati rice	Steam	0.0	139	69.1	1.582	3.390	0.0000	0.0000
24	White jasmine rice	Stir Fry	0.0	159	64.2	1.842	4.635	0.0000	0.0000
25	White jasmine rice	Steam	0.0	159	64.2	1.842	4.635	0.0000	0.0000
26	Yellow and egg noodle	Boil	0.0	352	66.0	1.684	4.995	0.0000	0.0000

Table S11: Summary of results for meat and meat products category (n=45)

	**Samples and Preparation**		**LC-MS/MS Analysis of Acrylamide**	**24-hour Dietary Recall Survey on Consumers (eaters only) from aged 15 to 92 years old**					
No.	Food Product	Cooking Method	Concentration (µg/kg)	Number of consumers surveyed	Mean Body Weight, (kg bw)	Consumption of mean consumers (g/kg bw)	Consumption of high consumers (P95) (g/kg bw)	Dietary exposure of mean consumers (µg/kg bw/day)	Dietary exposure of high consumers (P95) (µg/kg bw/day)
1	Beef bacon	Pan fry	5.7	3	60.0	0.205	0.269	0.0012	0.0015
2	Beef no fat	Boil	0.5	22	70.0	1.078	1.984	0.0005	0.0010
3	Beef no fat	Grill	6.5	22	70.0	1.078	1.984	0.0070	0.0128
4	Beef no fat	Pan fry	5.5	22	70.0	1.078	1.984	0.0059	0.0109

5	Beef no fat	Stir Fry	0.5	22	70.0	1.078	1.984	0.0005	0.0010
6	Beef no fat	Stew	0.5	22	70.0	1.078	1.984	0.0005	0.0010
7	Beef with fat	Boil	0.5	201	66.2	1.297	5.488	0.0006	0.0027
8	Beef with fat	Grill	2.3	201	66.2	1.297	5.488	0.0029	0.0123
9	Beef with fat	Pan fry	5.6	201	66.2	1.297	5.488	0.0073	0.0307
10	Beef with fat	Stir Fry	0.5	201	66.2	1.297	5.488	0.0006	0.0027
11	Beef with fat	Stew	0.5	201	66.2	1.297	5.488	0.0006	0.0027
12	Chicken	Boil	0.5	149	64.7	0.963	2.392	0.0005	0.0012
13	Chicken	Deep fry	5.7	29	67.5	1.391	2.876	0.0079	0.0164
14	Chicken	Roast	4.7	52	65.2	0.483	1.197	0.0023	0.0056
15	Chicken	Stir Fry	0.5	149	64.7	0.963	2.392	0.0005	0.0012
16	Chicken	Steam	3.6	149	64.7	0.963	2.392	0.0035	0.0086
17	Chicken	Soup	0.5	52	63.6	3.230	6.675	0.0016	0.0033
18	Chicken	Soup	0.5	52	63.6	3.230	6.675	0.0016	0.0033
19	Chicken ham bologna	RTE	0.5	81	64.4	0.392	0.857	0.0002	0.0004
20	Chicken nugget	Bake	14.3	61	68.4	0.711	1.231	0.0102	0.0176
21	Chicken nugget	Deep fry	20.1	61	68.4	0.711	1.231	0.0143	0.0247
22	Chicken sausage hot dog cocktail	Boil	3.6	76	68.6	0.560	1.307	0.0020	0.0047

23	Chicken sausage hot dog cocktail	Pan fry	0.5	76	68.6	0.560	1.307	0.0003	0.0007
24	Chinese sausage (eg. lup cheong)	Stir Fry	3.9	359	66.4	0.150	0.270	0.0006	0.0011
25	Duck	Boil	0.5	77	63.5	1.709	4.405	0.0009	0.0022
26	Duck	Braised	0.5	2	53.5	2.629	4.613	0.0013	0.0023
27	Duck	Roast	0.5	24	67.0	0.818	1.602	0.0004	0.0008
28	Mutton no fat	Boil	3.8	77	69.3	0.413	0.915	0.0016	0.0035
29	Mutton no fat	Stir Fry	13.3	77	69.3	0.413	0.915	0.0055	0.0122
30	Mutton no fat	Stew	13.0	77	69.3	0.413	0.915	0.0054	0.0119
31	Pork bacon	Pan fry	9.0	49	67.4	0.313	0.574	0.0028	0.0052
32	Pork ham bologna	RTE	0.5	46	64.2	0.290	0.506	0.0001	0.0003
33	Pork luncheon meat	Deep fry	0.5	77	66.2	0.880	2.367	0.0004	0.0012
34	Pork luncheon meat	No cooking	0.5	77	66.2	0.880	2.367	0.0004	0.0012
35	Pork luncheon meat	Pan fry	6.1	77	66.2	0.880	2.367	0.0054	0.0144
36	Pork luncheon meat	RTE	0.5	77	66.2	0.880	2.367	0.0004	0.0012
37	Pork meat ball	Boil	0.5	78	66.2	0.683	1.310	0.0003	0.0007
38	Pork no fat	Boil	0.5	648	65.7	0.720	2.011	0.0004	0.0010
39	Pork no fat	Roast	3.8	76	66.8	0.549	1.457	0.0021	0.0055
40	Pork no fat	Stir Fry	0.5	648	65.7	0.720	2.011	0.0004	0.0010

41	Pork organ	Boil	0.5	14	65.2	0.624	1.340	0.0003	0.0007
42	Pork with fat	Boil	0.5	294	64.9	0.749	2.351	0.0004	0.0012
43	Pork with fat	Roast	3.0	294	64.9	0.749	2.351	0.0022	0.0071
44	Pork with fat	Stir Fry	0.5	294	64.9	0.749	2.351	0.0004	0.0012
45	Prawn meat ball	Boil	0.5	9	70.4	0.524	0.928	0.0003	0.0005

Table S12: Summary of results for eggs and egg products category (n=11)

	**Samples and Preparation**		**LC-MS/MS Analysis of Acrylamide**	**24-hour Dietary Recall Survey on Consumers (eaters only) from aged 15 to 92 years old**					
No.	Food Product	Cooking Method	Concentration (µg/kg)	Number of consumers surveyed	Mean Body Weight, (kg bw)	Consumption of mean consumers (g/kg bw)	Consumption of high consumers (P95) (g/kg bw)	Dietary exposure of mean consumers (µg/kg bw/day)	Dietary exposure of high consumers (P95) (µg/kg bw/day)
1	Century egg	Boil	0.0	49	68.3	0.323	0.835	0.0000	0.0000
2	Century egg	RTE	4.6	49	68.3	0.323	0.835	0.0015	0.0038
3	Century egg	No cooking	0.0	49	68.3	0.323	0.835	0.0000	0.0000
4	Egg	Boil	0.0	392	64.3	0.662	1.591	0.0000	0.0000
5	Egg	Braised	0.0	775	66.0	0.477	1.064	0.0000	0.0000
6	Egg	Half-boil	0.0	392	64.3	0.662	1.591	0.0000	0.0000

7	Egg	Pan fry	0.0	455	65.8	0.590	1.512	0.0000	0.0000
8	Egg	Steam	0.0	775	66.0	0.477	1.064	0.0000	0.0000
9	Egg tofu	Boil	0.0	51	68.9	0.543	1.079	0.0000	0.0000
10	Egg tofu	Pan fry	0.0	51	68.9	0.543	1.079	0.0000	0.0000
11	Salted duck egg	Boil	0.0	57	64.8	0.253	0.681	0.0000	0.0000

Table S13: Summary of results for milk and dairy products category (n=15)

	**Samples and Preparation**		**LC-MS/MS Analysis of Acrylamide**	**24-hour Dietary Recall Survey on Consumers (eaters only) from aged 15 to 92 years old**					
No.	Food Product	Cooking Method	Concentration (µg/kg)	Number of consumers surveyed	Mean Body Weight, (kg bw)	Consumption of mean consumers (g/kg bw)	Consumption of high consumers (P95) (g/kg bw)	Dietary exposure of mean consumers (µg/kg bw/day)	Dietary exposure of high consumers (P95) (µg/kg bw/day)
1	Butter regular	No cooking	10.4	391	64.5	0.132	0.353	0.0014	0.0037
2	Cheese low fat	No cooking	0.0	74	63.2	0.314	0.754	0.0000	0.0000
3	Cheese regular	No cooking	0.0	74	63.2	0.314	0.754	0.0000	0.0000
4	Creamer	Reconstitute	0.0	80	68.2	0.101	0.275	0.0000	0.0000
5	Fermented milk	No cooking	0.0	62	62.1	1.558	2.845	0.0000	0.0000
6	Margarine	No cooking	0.0	66	68.5	0.097	0.235	0.0000	0.0000

7	Milk condensed	No cooking	0.0	232	66.7	0.193	0.484	0.0000	0.0000
8	Milk low fat	No cooking	0.0	11	57.6	3.067	6.102	0.0000	0.0000
9	Milk powdered low fat	Reconstitute	0.0	4	59.0	0.139	0.188	0.0000	0.0000
10	Milk powdered regular	Reconstitute	0.0	122	61.2	0.297	1.166	0.0000	0.0000
11	Milk powdered skimmed	Reconstitute	0.0	4	59.0	0.139	0.188	0.0000	0.0000
12	Milk regular	No cooking	0.0	358	64.8	2.501	6.716	0.0000	0.0000
13	Milk skimmed	No cooking	0.0	82	65.9	2.689	6.022	0.0000	0.0000
14	Yoghurt low fat	No cooking	0.0	68	63.9	2.244	4.863	0.0000	0.0000
15	Yoghurt regular	No cooking	0.0	68	63.9	2.244	4.863	0.0000	0.0000

Table S14: Summary of results for fish and seafood category (n=62)

No.	**Samples and Preparation**		**LC-MS/MS Analysis of Acrylamide**	**24-hour Dietary Recall Survey on Consumers (eaters only) from aged 15 to 92 years old**					
	Food Product	Cooking Method	Concentration (µg/kg)	Number of consumers surveyed	Mean Body Weight, (kg bw)	Consumption of mean consumers (g/kg bw)	Consumption of high consumers (P95) (g/kg bw)	Dietary exposure of mean consumers (µg/kg bw/day)	Dietary exposure of high consumers (P95) (µg/kg bw/day)

1	Anchovy	Deep fry	7.6	76	60.0	0.055	0.149	0.0004	0.0011
2	Anchovy	Pan fry	3.1	76	60.0	0.055	0.149	0.0002	0.0005
3	Anchovy	Soup	1.0	76	60.0	0.055	0.149	0.0001	0.0002
4	Anchovy	Soup	0.0	76	60.0	0.055	0.149	0.0000	0.0000
5	Canned sardine	Stir Fry	0.0	11	73.6	0.530	0.970	0.0000	0.0000
6	Canned sardine	Stew	0.0	11	73.6	0.530	0.970	0.0000	0.0000
7	Canned tuna	No cooking	0.0	51	66.6	0.830	1.796	0.0000	0.0000
8	Catfish	Deep fry	0.0	11	66.2	1.607	4.857	0.0000	0.0000
9	Clam	Boil	3.9	17	64.2	0.930	2.476	0.0036	0.0097
10	Clam	Stir Fry	0.0	17	64.2	0.930	2.476	0.0000	0.0000
11	COCKLE	Boil	0.0	101	68.1	0.135	0.262	0.0000	0.0000
12	COCKLE	RTE	0.0	101	68.1	0.135	0.262	0.0000	0.0000
13	COCKLE	Stir Fry	0.0	101	68.1	0.135	0.262	0.0000	0.0000
14	Crab	Boil	0.0	25	69.2	0.929	1.941	0.0000	0.0000
15	Crab	Stir Fry	0.0	25	69.2	0.929	1.941	0.0000	0.0000
16	Fish ball fish cake and related product	Boil	0.0	305	65.0	0.382	0.799	0.0000	0.0000
17	Fish Head boiled	Soup	0.0	34	63.2	4.066	16.421	0.0000	0.0000

18	Fish Head boiled	Soup	0.0	34	63.2	4.066	16.421	0.0000	0.0000
19	Fish nugget and related product	Bake	0.0	1	56.0	0.136	0.136	0.0000	0.0000
20	Fish nugget and related product	Deep fry	0.0	23	65.7	0.584	1.095	0.0000	0.0000
21	Fish roe	RTE	0.0	2	74.5	0.106	0.126	0.0000	0.0000
22	GROUPER	Steam	0.0	75	62.0	0.857	3.332	0.0000	0.0000
23	Kuning and related fishes	Deep fry	0.0	11	69.0	0.975	2.231	0.0000	0.0000
24	Lobster / crayfish	Boil	0.0	4	55.8	1.171	2.496	0.0000	0.0000
25	Lobster / crayfish	Stir Fry	0.0	4	55.8	1.171	2.496	0.0000	0.0000
26	Mackerel and related fishes (e.g. batang)	Pan fry	0.0	32	66.3	0.749	1.749	0.0000	0.0000
27	Mussel	Stir Fry	0.0	6	69.5	0.120	0.285	0.0000	0.0000
28	Mussel	Steam	0.0	6	69.5	0.120	0.285	0.0000	0.0000
29	Mussel	Steam	0.0	6	69.5	0.120	0.285	0.0000	0.0000
30	Orh luak	Pan fry	11.2	20	62.2	0.175	0.403	0.0020	0.0045
31	Oyster	Boil	0.0	20	62.2	0.175	0.403	0.0000	0.0000
32	Oyster	RTE	0.0	20	62.2	0.175	0.403	0.0000	0.0000
33	Oyster	Stir Fry	0.0	20	62.2	0.175	0.403	0.0000	0.0000
34	Prawn / shrimp	Boil	0.0	2	57.0	1.084	1.406	0.0000	0.0000

35	Prawn / shrimp	Deep fry	0.0	2	57.0	1.084	1.406	0.0000	0.0000
36	Prawn / shrimp	Stir Fry	0.0	2	57.0	1.084	1.406	0.0000	0.0000
37	Prawn / shrimp	Soup	0.0	2	57.0	1.084	1.406	0.0000	0.0000
38	Prawn / shrimp	Soup	0.0	2	57.0	1.084	1.406	0.0000	0.0000
39	Salmon	Pan fry	0.0	147	63.3	1.216	3.514	0.0000	0.0000
40	Scallop	Boil	0.0	36	67.4	0.728	1.533	0.0000	0.0000
41	Scallop	RTE	0.0	36	67.4	0.728	1.533	0.0000	0.0000
42	Salted fish and related product	Deep fry	0.0	90	64.6	0.039	0.090	0.0000	0.0000
43	Salted fish and related product	Stir Fry	0.0	90	64.6	0.039	0.090	0.0000	0.0000
44	Scallop	Stir Fry	0.0	36	67.4	0.728	1.533	0.0000	0.0000
45	Scallop	Soup	0.0	36	67.4	0.728	1.533	0.0000	0.0000
46	Scallop	Soup	0.0	36	67.4	0.728	1.533	0.0000	0.0000
47	Sea cucumber	Boil	0.0	5	67.8	2.292	7.681	0.0000	0.0000
48	SEABASS	Steam	0.0	21	70.7	1.512	3.142	0.0000	0.0000
49	SNAPPER	Steam	0.0	16	64.6	1.248	3.680	0.0000	0.0000
50	Squid / cuttlefish	Boil	0.0	128	65.5	0.577	1.695	0.0000	0.0000
51	Squid / cuttlefish	Stir Fry	0.0	128	65.5	0.577	1.695	0.0000	0.0000
52	Squid ball and related product	Boil	0.0	5	73.4	0.480	0.868	0.0000	0.0000

53	Threadfin (ngor he)	Steam	0.0	21	63.2	0.884	1.421	0.0000	0.0000
54	Trout / Cod	Boil	0.0	26	65.6	0.560	2.103	0.0000	0.0000
55	Trout / Cod	Deep fry	0.0	26	65.6	0.560	2.103	0.0000	0.0000
56	Trout / Cod	Steam	25.3	26	65.6	0.560	2.103	0.0142	0.0532
57	Trout / Cod	Steam	0.0	26	65.6	0.560	2.103	0.0000	0.0000
58	Trout / Cod	Stew	25.4	26	65.6	0.560	2.103	0.0142	0.0533
59	Tuna	Braised	0.0	7	60.3	0.557	1.452	0.0000	0.0000
60	Tuna	Pan fry	1.7	7	60.3	0.557	1.452	0.0009	0.0024
61	Salted fish and related product	Stir Fry	20.0	90	64.6	0.039	0.090	0.0008	0.0018
62	Salted fish and related product	Deep fry	5.0	90	64.6	0.039	0.090	0.0002	0.0005

Table S15: Summary of results for fats and oils category (n=4)

	**Samples and Preparation**		**LC-MS/MS Analysis of Acrylamide**	**24-hour Dietary Recall Survey on Consumers (eaters only) from aged 15 to 92 years old**					
No.	Food Product	Cooking Method	Concentration (µg/kg)	Number of consumers surveyed	Mean Body Weight, (kg bw)	Consumption of mean consumers (g/kg bw)	Consumption of high consumers (P95) (g/kg bw)	Dietary exposure of mean consumers	Dietary exposure of high consumers

								(µg/kg bw/day)	(P95) (µg/kg bw/day)
1	Olive oil	No cooking	0.0	110	64.7	0.135	0.332	0.0000	0.0000
2	Vegetable oil	Deep fry	0.0	32	65.4	0.238	0.765	0.0000	0.0000
3	Vegetable oil	Stir Fry	0.0	32	65.4	0.238	0.765	0.0000	0.0000
4	Vegetable oil	No cooking	0.0	32	65.4	0.238	0.765	0.0000	0.0000

Table S16: Summary of results for sauces and condiments category (n=25)

	**Samples and Preparation**		**LC-MS/MS Analysis of Acrylamide**	**24-hour Dietary Recall Survey on Consumers (eaters only) from aged 15 to 92 years old**					
No.	Food Product	Cooking Method	Concentration (µg/kg)	Number of consumers surveyed	Mean Body Weight, (kg bw)	Consumption of mean consumers (g/kg bw)	Consumption of high consumers (P95) (g/kg bw)	Dietary exposure of mean consumers (µg/kg bw/day)	Dietary exposure of high consumers (P95) (µg/kg bw/day)
1	Thousand island sauce	No cooking	0.5	2	58.5	0.145	0.145	0.0001	0.0001
2	Cheese sauce	No cooking	0.5	50	65.4	0.206	0.496	0.0001	0.0002
3	Chilli powder	No cooking	293.1	164	66.8	0.018	0.059	0.0053	0.0174
4	Chilli sauce	No cooking	17.7	869	66.1	0.142	0.537	0.0025	0.0095

5	Cream sauce	Boil	4.4	16	68.9	0.516	1.283	0.0023	0.0056
6	Fish sauce	No cooking	0.5	448	65.7	0.025	0.081	0.0000	0.0000
7	Mayonnaise	No cooking	5.7	133	66.4	0.129	0.314	0.0007	0.0018
8	MSG	No cooking	0.5	35	64.2	0.019	0.027	0.0000	0.0000
9	Mustard sauce	No cooking	6.1	1	84.0	0.031	0.031	0.0002	0.0002
10	Oil sesame	No cooking	15.6	930	65.9	0.033	0.115	0.0005	0.0018
11	Oyster sauce	No cooking	0.5	429	66.0	0.081	0.213	0.0000	0.0001
12	Pepper black	No cooking	403.8	226	67.4	0.010	0.031	0.0040	0.0126
13	Pepper white powder	No cooking	16.5	814	65.3	0.012	0.035	0.0002	0.0006
14	Salt	No cooking	0.5	1310	65.7	0.024	0.095	0.0000	0.0000
15	Satay sauce	Boil	42.8	27	67.0	1.931	3.894	0.0827	0.1667
16	Sesame seed	No cooking	26.0	16	63.9	0.040	0.072	0.0010	0.0019
17	Soy sauce	No cooking	0.5	756	65.4	0.086	0.240	0.0000	0.0001
18	Soy sauce dark	No cooking	0.5	756	65.4	0.086	0.240	0.0000	0.0001
19	Soy sauce light	Boil	6.5	756	65.4	0.086	0.240	0.0006	0.0016
20	Sambal chilli/belacan	Stir Fry	111.5	148	65.9	0.753	3.326	0.0840	0.3708
21	Soy sauce light	RTE	5.5	756	65.4	0.086	0.240	0.0005	0.0013
22	Sugar white	No cooking	0.5	406	66.3	0.084	0.246	0.0000	0.0001
23	Teriyaki sauce	No cooking	0.5	37	62.6	0.194	0.424	0.0001	0.0002
24	Tomato sauce	No cooking	10.5	251	66.5	0.268	0.858	0.0028	0.0090

25	Vinegar	No cooking	0.5	118	66.3	0.101	0.292	0.0001	0.0001

Table S17: Summary of results for beverages category (excluding coffee and coffee substitutes) (n=10)

	**Samples and Preparation**		**LC-MS/MS Analysis of Acrylamide**	**24-hour Dietary Recall Survey on Consumers (eaters only) from aged 15 to 92 years old**					
No.	Food Product	Cooking Method	Concentration (µg/kg)	Number of consumers surveyed	Mean Body Weight, (kg bw)	Consumption of mean consumers (g/kg bw)	Consumption of high consumers (P95) (g/kg bw)	Dietary exposure of mean consumers (µg/kg bw/day)	Dietary exposure of high consumers (P95) (µg/kg bw/day)
1	Alcohol Beer	RTE	3.6	64	68.1	2.917	7.575	0.0105	0.0273
2	Alcohol Red Wine	No cooking	0.0	53	64.4	1.685	4.338	0.0000	0.0000
3	Alcohol White Wine	No cooking	0.0	8	62.0	3.665	9.285	0.0000	0.0000
4	Non-carbonated sweetened beverage	No cooking	7.9	18	66.6	2.331	4.482	0.0184	0.0354
5	Tap water	No cooking	0.0	2014	65.1	29.463	53.876	0.0000	0.0000
6	Green tea and chinese tea	Brew	0.0	17	61.7	1.650	2.925	0.0000	0.0000
7	Juice drink (manufactured	No cooking	0.0	30	66.2	2.158	4.231	0.0000	0.0000

	excluding freshly prepared juices)								
8	Milk tea	No cooking	0.0	13	62.0	2.613	4.273	0.0000	0.0000
9	Red (english) tea	Brew	0.0	3	65.7	1.952	2.342	0.0000	0.0000
10	Sugarcane juice	No cooking	0.0	19	60.3	2.003	2.922	0.0000	0.0000

Table S18: Summary of results for bakery products category (excluding biscuits and wafers) (n=6)

No.	Samples and Preparation		LC-MS/MS Analysis of Acrylamide	24-hour Dietary Recall Survey on Consumers (eaters only) from aged 15 to 92 years old					
	Food Product	Cooking Method	Concentration (µg/kg)	Number of consumers surveyed	Mean Body Weight, (kg bw)	Consumption of mean consumers (g/kg bw)	Consumption of high consumers (P95) (g/kg bw)	Dietary exposure of mean consumers (µg/kg bw/day)	Dietary exposure of high consumers (P95) (µg/kg bw/day)
1	Savoury bun (bao)	No cooking	10.6	4	63.5	0.582	1.082	0.0062	0.0115
2	Bun custard	No cooking	10.9	24	63.8	0.833	1.800	0.0091	0.0196
3	Pancake	No cooking	0.5	4	57.8	0.429	0.625	0.0002	0.0003
4	Sweet bun (bao)	No cooking	10.9	46	66.0	0.648	1.329	0.0071	0.0145
5	WHITE BREAD	No cooking	12.3	658	64.9	0.647	1.321	0.0080	0.0163
6	Wholemeal bread	No cooking	14.4	362	64.8	0.304	0.669	0.0044	0.0096

Table S19: Summary of results for composite foods category (n=12)

No.	Samples and Preparation		LC-MS/MS Analysis of Acrylamide	24-hour Dietary Recall Survey on Consumers (eaters only) from aged 15 to 92 years old					
	Food Product	Cooking Method	Concentration (μg/kg)	Number of consumers surveyed	Mean Body Weight, (kg bw)	Consumption of mean consumers (g/kg bw)	Consumption of high consumers (P95) (g/kg bw)	Dietary exposure of mean consumers (μg/kg bw/day)	Dietary exposure of high consumers (P95) (μg/kg bw/day)
1	Carrot cake	Pan fry	7.8	68	65.7	1.917	4.093	0.0150	0.0319
2	Curry gravy (fish)	Boil	57.6	7	66.6	1.030	1.959	0.0593	0.1128
3	Meat dumpling	RTE	0.5	2	69.5	1.215	1.776	0.0006	0.0009
4	Curry gravy (meat)	Boil	30.1	7	70.1	3.076	8.226	0.0926	0.2476
5	Dhal	Boil	0.5	68	64.2	3.648	8.340	0.0018	0.0042
6	Idli	No cooking	0.5	21	68.3	2.263	3.947	0.0011	0.0020
7	Kimchi	No cooking	0.5	34	61.4	0.604	2.568	0.0003	0.0013
8	Mushroom soup (canned instant prepared)	Boil	3.8	31	62.3	2.353	5.182	0.0089	0.0197
9	Pizza	No cooking	0.5	12	69.9	0.995	2.478	0.0005	0.0012

10	Putu mayam	Steam	0.5	2	62.5	0.793	1.134	0.0004	0.0006
11	You tiao - w alum	Deep fry	55.4	60	66.6	0.492	1.100	0.0272	0.0609
12	You tiao (w/o alum)	Deep fry	54.4	60	66.6	0.492	1.100	0.0267	0.0598

Foods commonly consumed between the main meals tested for the occurrence of acrylamide

Table S20 to S22 were ready-to-eat foods selected on the basis that they are commonly consumed outside of the main meals in Singapore. Their brands have been anomalised to provide confidentiality of the manufacturers. There are 3 food categories with a total of 386 food samples tested for their acrylamide concentration (µg/kg or ppb) using LC-MS/MS with a limit of detection (LOD) of 1.0 µg/kg. Those samples not detected with acrylamide (i.e. concentration below the LOD) are treated according to the WHO recommendation on the evaluation of low-level contaminant of food. The concentrations below LOD were set at 0 when the food category contains more than 60% of samples not detected with acrylamide; while the concentration below LOD were set at half the LOD when the food category contained less than 60% of samples not detected with acrylamide. The food consumption data of Singapore population were obtained from 24-hour dietary recall surveys for consumers from age 15 to 92 years old. The consumption amount (g/kg body weight or bw) of mean and high consumers (P95) were determined. The dietary exposure of acrylamide from each food product was calculated by multiplying the concentration (µg/kg) and consumption amount (g/kg bw) together with a unit conversion factor (10^{-3}).

Table S20: Summary of results for RTE savouries category (crackers and chips, biscuits and wafers, cereal-based snack bars, dried snack food (n=295)

	**Food Products and Information**			**LC-MS/MS Analysis of Acrylamide**	**24-hour Dietary Recall Survey on Consumers (eaters only) from aged 15 to 92 years old**					
No.	Food Product	Brand name (coded)	Country of origin	Concentration (µg/kg)	Number of consumers surveyed	Mean Body Weight, (kg bw)	Consumption of mean consumers (g/kg bw)	Consumption of high consumers (P95) (g/kg bw)	Dietary exposure of mean consumers (µg/kg bw/day)	Dietary exposure of high consumers (P95) (µg/kg bw/day)

1	Potato chips or crackers	F-127	UNITED STATES	1535.4	81	63.9	0.288	0.751	0.4417	1.1530
2	Potato chips or crackers	W-374	MALAYSIA	1336.8	81	63.9	0.288	0.751	0.3846	1.0039
3	Potato chips or crackers	T-285	MALAYSIA	1135.8	81	63.9	0.288	0.751	0.3267	0.8529
4	Potato chips or crackers	C-408	MALAYSIA	848.9	81	63.9	0.288	0.751	0.2442	0.6375
5	Potato chips or crackers	C-408	MALAYSIA	725.9	81	63.9	0.288	0.751	0.2088	0.5451
6	Potato chips or crackers	R-220	AUSTRALIA	693.9	81	63.9	0.288	0.751	0.1996	0.5211
7	Potato chips or crackers	L-347	UNITED STATES	329.6	81	63.9	0.288	0.751	0.0948	0.2475
8	Potato chips or crackers	P-247	MALAYSIA	270.6	81	63.9	0.288	0.751	0.0778	0.2032
9	Potato chips or crackers	T-51	AUSTRALIA	256.2	81	63.9	0.288	0.751	0.0737	0.1924
10	Potato chips or crackers	L-107	CHINA	201.2	81	63.9	0.288	0.751	0.0579	0.1511
11	Potato chips or crackers	L-347	UNITED STATES	181.4	81	63.9	0.288	0.751	0.0522	0.1362
12	Potato chips or crackers	T-35	MALAYSIA	81.8	81	63.9	0.288	0.751	0.0235	0.0614

13	Potato chips or crackers	C-408	MALAYSIA	1611.1	81	63.9	0.288	0.751	0.4635	1.2099
14	Potato chips or crackers	F-60	MALAYSIA	1182	81	63.9	0.288	0.751	0.3400	0.8876
15	Potato chips or crackers	F-60	MALAYSIA	999.7	81	63.9	0.288	0.751	0.2876	0.7507
16	Potato chips or crackers	F-60	MALAYSIA	950.1	81	63.9	0.288	0.751	0.2733	0.7135
17	Potato chips or crackers	C-408	MALAYSIA	833.6	81	63.9	0.288	0.751	0.2398	0.6260
18	Potato chips or crackers	J-314	CHINA	785.8	81	63.9	0.288	0.751	0.2260	0.5901
19	Potato chips or crackers	T-274	MALAYSIA	633.7	81	63.9	0.288	0.751	0.1823	0.4759
20	Potato chips or crackers	F-60	MALAYSIA	552.5	81	63.9	0.288	0.751	0.1589	0.4149
21	Potato chips or crackers	J-314	MALAYSIA	552.1	81	63.9	0.288	0.751	0.1588	0.4146
22	Potato chips or crackers	P-247	MALAYSIA	491.8	81	63.9	0.288	0.751	0.1415	0.3693
23	Potato chips or crackers	P-336	TAIWAN	468.6	81	63.9	0.288	0.751	0.1348	0.3519
24	Potato chips or crackers	T-343	Unknown	447.9	81	63.9	0.288	0.751	0.1288	0.3364

25	Potato chips or crackers	A-69	AUSTRALIA	411.7	81	63.9	0.288	0.751	0.1184	0.3092
26	Potato chips or crackers	O-142	NEW ZEALAND	278.5	81	63.9	0.288	0.751	0.0801	0.2091
27	Potato chips or crackers	L-347	UNITED STATES	247.6	81	63.9	0.288	0.751	0.0712	0.1859
28	Potato chips or crackers	L-347	UNITED STATES	182.4	81	63.9	0.288	0.751	0.0525	0.1370
29	Potato chips or crackers	R-287	UNITED STATES	160	81	63.9	0.288	0.751	0.0460	0.1202
30	Potato chips or crackers	B-275	UNITED KINGDOM	667.8	81	63.9	0.288	0.751	0.1921	0.5015
31	Potato chips or crackers	P-247	MALAYSIA	2095.8	81	63.9	0.288	0.751	0.6029	1.5739
32	Potato chips or crackers	H-453	CHINA	993.3	81	63.9	0.288	0.751	0.2857	0.7459
33	Potato chips or crackers	T-69	JAPAN	1148.3	81	63.9	0.288	0.751	0.3303	0.8623
34	Potato chips or crackers	C-408	JAPAN	784.7	81	63.9	0.288	0.751	0.2257	0.5893
35	Potato chips or crackers	C-408	JAPAN	1965.5	81	63.9	0.288	0.751	0.5654	1.4760
36	Potato chips or crackers	Y-425	JAPAN	335.8	81	63.9	0.288	0.751	0.0966	0.2522

37	Crackers (fruit)	H-221	INDONESIA	2866.5	15	64.6	0.412	0.676	1.1799	1.9381
38	Crackers (Wheat flour)	H-453	MALAYSIA	600.6	15	64.6	0.412	0.676	0.2472	0.4061
39	Crackers (Wheat flour)	J-349	MALAYSIA	459.7	15	64.6	0.412	0.676	0.1892	0.3108
40	Crackers (Rice & Dhall flour)	D-364	MALAYSIA	458.4	15	64.6	0.412	0.676	0.1887	0.3099
41	Popcorn	P-470	KOREA, REPUBLIC OF	375.3	5	73.6	0.125	0.229	0.0468	0.0859
42	Crackers (Rice)	B-118	SINGAPORE	371	15	64.6	0.412	0.676	0.1527	0.2508
43	Crackers (Wheat flour)	S-103	SINGAPORE	332	6	57.3	0.820	1.455	0.2721	0.4830
44	Crackers (Rice & Dhall flour)	B-496	INDIA	321.8	15	64.6	0.412	0.676	0.1325	0.2176
45	Crackers (Wheat flour)	H-351	MALAYSIA	304.2	15	64.6	0.412	0.676	0.1252	0.2057
46	Crackers (Rice & Dhall flour)	M-490	MALAYSIA	294.7	15	64.6	0.412	0.676	0.1213	0.1993
47	Crackers (Rice)	S-353	JAPAN	263.4	15	64.6	0.412	0.676	0.1084	0.1781
48	Crackers (Corn)	D-116	TAIWAN	238.8	1	95.0	0.474	0.474	0.1131	0.1131
49	Crackers (Rice)	S-353	JAPAN	222.1	15	64.6	0.412	0.676	0.0914	0.1502
50	Crackers (Wheat flour)	C-408	THAILAND	206.2	6	57.3	0.820	1.455	0.1690	0.3000

51	Crackers (Corn)	M-97	CHINA	164.3	1	95.0	0.474	0.474	0.0778	0.0778
52	Crackers (Corn)	R-120	JAPAN	157.9	1	95.0	0.474	0.474	0.0748	0.0748
53	Crackers (Wheat flour)	C-75	MALAYSIA	105.6	15	64.6	0.412	0.676	0.0435	0.0714
54	Crackers (fruit)	T-332	INDIA	105.1	15	64.6	0.412	0.676	0.0433	0.0711
55	Crackers (Corn)	T-274	MALAYSIA	104	1	95.0	0.474	0.474	0.0493	0.0493
56	Crackers (fruit)	B-265	UNITED STATES	96.2	15	64.6	0.412	0.676	0.0396	0.0650
57	Crackers (fruit)	F-60	SINGAPORE	94.1	15	64.6	0.412	0.676	0.0387	0.0636
58	Crackers (Sorghum)	A-327	UNITED STATES	92.5	15	64.6	0.412	0.676	0.0381	0.0625
59	Crackers (Wheat flour)	C-408	THAILAND	91.9	6	57.3	0.820	1.455	0.0753	0.1337
60	Crackers (Cassava)	M-351	INDONESIA	75.7	15	64.6	0.412	0.676	0.0312	0.0512
61	Crackers (Tapioca)	F-60	SINGAPORE	72.5	15	64.6	0.412	0.676	0.0298	0.0490
62	Crackers (Tapioca)	B-446	SINGAPORE	69.4	15	64.6	0.412	0.676	0.0286	0.0469
63	Crackers (Tapioca)	F-60	SINGAPORE	69.2	6	57.3	0.820	1.455	0.0567	0.1007
64	Crackers (Wheat flour)	J-74	MALAYSIA	67.8	15	64.6	0.412	0.676	0.0279	0.0458
65	Crackers (Rice)	U-28	JAPAN	66.8	15	64.6	0.412	0.676	0.0275	0.0452
66	Crackers (Rice)	U-235	MALAYSIA	62.7	15	64.6	0.412	0.676	0.0258	0.0424

67	Crackers (Wheat flour)	P-26	KOREA, REPUBLIC OF	60.4	15	64.6	0.412	0.676	0.0249	0.0408
68	Crackers (Corn)	C-277	KOREA, REPUBLIC OF	54.7	1	95.0	0.474	0.474	0.0259	0.0259
69	Crackers (Cassava)	M-351	INDONESIA	51.2	15	64.6	0.412	0.676	0.0211	0.0346
70	Crackers (Rice)	S-353	JAPAN	49.3	15	64.6	0.412	0.676	0.0203	0.0333
71	Crackers (Rice)	W-368	THAILAND	47.9	15	64.6	0.412	0.676	0.0197	0.0324
72	Crackers (Corn)	M-419	MALAYSIA	45.9	1	95.0	0.474	0.474	0.0217	0.0217
73	Crackers (fruit)	-	MALAYSIA	44.9	15	64.6	0.412	0.676	0.0185	0.0304
74	Crackers (Corn)	M-419	MALAYSIA	38.8	1	95.0	0.474	0.474	0.0184	0.0184
75	Crackers (Rice & Dhall flour)	A-489	MALAYSIA	38.6	15	64.6	0.412	0.676	0.0159	0.0261
76	Crackers (Corn)	G-127	KOREA, REPUBLIC OF	38.5	1	95.0	0.474	0.474	0.0182	0.0182
77	Crackers (Wheat flour)	P-375	UNITED STATES	35.3	15	64.6	0.412	0.676	0.0145	0.0239
78	Crackers (Seafood)	C-194	SINGAPORE	35.1	1	65.0	0.142	0.142	0.0050	0.0050
79	Crackers (Rice)	S-353	JAPAN	34.8	15	64.6	0.412	0.676	0.0143	0.0235
80	Crackers (Rice & Dhall flour)	B-496	MALAYSIA	32.4	15	64.6	0.412	0.676	0.0133	0.0219
81	Crackers (Rice)	C-426	THAILAND	32.3	15	64.6	0.412	0.676	0.0133	0.0218

82	Crackers (Wheat flour)	B-435	KOREA, REPUBLIC OF	32	15	64.6	0.412	0.676	0.0132	0.0216
83	Crackers (Wheat flour)	K-162	GERMANY	31.9	15	64.6	0.412	0.676	0.0131	0.0216
84	Crackers (Rice & Dhall flour)	B-496	MALAYSIA	30.7	15	64.6	0.412	0.676	0.0126	0.0208
85	Crackers (Tapioca)	-	MALAYSIA	29.1	15	64.6	0.412	0.676	0.0120	0.0197
86	Crackers (Tapioca)	M-111	Unknown	27.5	15	64.6	0.412	0.676	0.0113	0.0186
87	Crackers (Seafood)	T-402	SINGAPORE	24.2	1	65.0	0.142	0.142	0.0034	0.0034
88	Crackers (Rice & Dhall flour)	F-60	SINGAPORE	24.1	15	64.6	0.412	0.676	0.0099	0.0163
89	Crackers (Tapioca)	M-468	INDIA	22.8	15	64.6	0.412	0.676	0.0094	0.0154
90	Crackers (Rice)	W-368	THAILAND	22.7	15	64.6	0.412	0.676	0.0093	0.0153
91	Crackers (Wheat flour)	T-204	IRELAND	20.3	15	64.6	0.412	0.676	0.0084	0.0137
92	Crackers (Seafood)	-	SINGAPORE	18.3	1	65.0	0.142	0.142	0.0026	0.0026
93	Crackers (Rice & Dhall flour)	V-385	MALAYSIA	16.4	15	64.6	0.412	0.676	0.0068	0.0111
94	Crackers (Wheat flour)	M-409	SINGAPORE	14.4	15	64.6	0.412	0.676	0.0059	0.0097
95	Crackers (Seafood)	-	SINGAPORE	13.1	1	65.0	0.142	0.142	0.0019	0.0019
96	Crackers (Tapioca)	Y-373	SINGAPORE	13	15	64.6	0.412	0.676	0.0054	0.0088

97	Crackers (Rice)	T-126	JAPAN	11.9	15	64.6	0.412	0.676	0.0049	0.0080
98	Crackers (Rice)	W-342	CHINA	11.4	15	64.6	0.412	0.676	0.0047	0.0077
99	Crackers (Seafood)	W-48	THAILAND	11.2	1	65.0	0.142	0.142	0.0016	0.0016
100	Crackers (Corn)	B-473	JAPAN	10.6	1	95.0	0.474	0.474	0.0050	0.0050
101	Crackers (Tapioca)	C-195	KOREA, REPUBLIC OF	10.6	15	64.6	0.412	0.676	0.0044	0.0072
102	Crackers (Tapioca)	Y-373	MALAYSIA	0.5	15	64.6	0.412	0.676	0.0002	0.0003
103	Crackers (Tapioca)	K-20	MALAYSIA	0.5	15	64.6	0.412	0.676	0.0002	0.0003
104	Crackers (Tapioca)	K-20	MALAYSIA	0.5	15	64.6	0.412	0.676	0.0002	0.0003
105	Crackers (Tapioca)	B-434	INDONESIA	0.5	15	64.6	0.412	0.676	0.0002	0.0003
106	Crackers (Tapioca)	K-20	SINGAPORE	0.5	15	64.6	0.412	0.676	0.0002	0.0003
107	Crackers (Tapioca)	Y-373	MALAYSIA	0.5	15	64.6	0.412	0.676	0.0002	0.0003
108	Crackers (Seafood)	B-161	CHINA	0.5	1	65.0	0.142	0.142	0.0001	0.0001
109	Crackers (Tapioca)	S-436	INDIA	0.5	15	64.6	0.412	0.676	0.0002	0.0003
110	Crackers (fruit)	-	INDONESIA	0.5	15	64.6	0.412	0.676	0.0002	0.0003
111	Crackers (Tapioca)	K-20	Unknown	0.5	6	57.3	0.820	1.455	0.0004	0.0007
112	Crackers (Tapioca)	-	MALAYSIA	0.5	15	64.6	0.412	0.676	0.0002	0.0003
113	Crackers (Wheat flour)	P-26	KOREA, REPUBLIC OF	0.5	15	64.6	0.412	0.676	0.0002	0.0003
114	Crackers (Tapioca)	B-91	MALAYSIA	0.5	15	64.6	0.412	0.676	0.0002	0.0003

115	Crackers (fruit)	K-20	SINGAPORE	0.5	15	64.6	0.412	0.676	0.0002	0.0003
116	Crackers (Seafood)	M-169	THAILAND	0.5	6	57.3	0.820	1.455	0.0004	0.0007
117	Crackers (fruit)	F-60	SINGAPORE	0.5	15	64.6	0.412	0.676	0.0002	0.0003
118	Crackers (Wheat flour)	F-60	SINGAPORE	0.5	6	57.3	0.820	1.455	0.0004	0.0007
119	Crackers (Wheat flour)	F-60	SINGAPORE	0.5	15	64.6	0.412	0.676	0.0002	0.0003
120	Crackers (Wheat flour)	T-402	SINGAPORE	0.5	15	64.6	0.412	0.676	0.0002	0.0003
121	Crackers (Rice)	T-276	THAILAND	89.3	15	64.6	0.412	0.676	0.0368	0.0604
122	Crackers (Taro)	M-322	THAILAND	0.5	15	64.6	0.412	0.676	0.0002	0.0003
123	Crackers (fruit)	J-287	THAILAND	137.4	15	64.6	0.412	0.676	0.0566	0.0929
124	Crackers (Seafood)	M-322	THAILAND	0.5	1	65.0	0.142	0.142	0.0001	0.0001
125	Crackers (Fish skin)	S-84	SINGAPORE	24.7	1	65.0	0.142	0.142	0.0035	0.0035
126	Crackers (Seafood)	F-60	SINGAPORE	0.5	6	57.3	0.820	1.455	0.0004	0.0007
127	Crackers (Fish skin)	C-194	SINGAPORE	28.7	1	65.0	0.142	0.142	0.0041	0.0041
128	Crackers (Cassava)	M-351	INDONESIA	0.5	15	64.6	0.412	0.676	0.0002	0.0003
129	Crackers (Fish skin)	I-33	SINGAPORE	11.8	1	65.0	0.142	0.142	0.0017	0.0017
130	Crackers (Cassava)	U-262	MALAYSIA	30.1	15	64.6	0.412	0.676	0.0124	0.0204
131	Crackers (Tortilla)	D-116	CHINA	57.5	15	64.6	0.412	0.676	0.0237	0.0389
132	Crackers (Tortilla)	M-240	INDONESIA	135.6	15	64.6	0.412	0.676	0.0558	0.0917

133	Crackers (Seafood)	C-408	THAILAND	98.9	6	57.3	0.820	1.455	0.0811	0.1439
134	Crackers (Seafood)	N-205	MALAYSIA	0.5	6	57.3	0.820	1.455	0.0004	0.0007
135	Crackers (Cassava)	M-351	INDONESIA	47.8	15	64.6	0.412	0.676	0.0197	0.0323
136	Crackers (Tapioca)	K-20	SINGAPORE	23.8	15	64.6	0.412	0.676	0.0098	0.0161
137	Crackers (Cassava)	M-351	INDONESIA	87.2	15	64.6	0.412	0.676	0.0359	0.0590
138	Crackers (Seafood)	S-479	SINGAPORE	0.5	6	57.3	0.820	1.455	0.0004	0.0007
139	Crackers (Seafood)	S-6	Unknown	0.5	6	57.3	0.820	1.455	0.0004	0.0007
140	Crackers (Rice)	B-449	THAILAND	0.5	15	64.6	0.412	0.676	0.0002	0.0003
141	Crackers (Seafood)	S-6	SINGAPORE	0.5	1	65.0	0.142	0.142	0.0001	0.0001
142	Crackers (Seafood)	S-6	SINGAPORE	0.5	6	57.3	0.820	1.455	0.0004	0.0007
143	Crackers (fruit)	B-150	MALAYSIA	51.7	15	64.6	0.412	0.676	0.0213	0.0350
144	Crackers (Tapioca)	D-498	MALAYSIA	51.3	15	64.6	0.412	0.676	0.0211	0.0347
145	Crackers (Seafood)	C-408	THAILAND	132.7	6	57.3	0.820	1.455	0.1088	0.1931
146	Crackers (Seafood)	G-186	MALAYSIA	0.5	6	57.3	0.820	1.455	0.0004	0.0007
147	Crackers (Seafood)	M-477	MALAYSIA	0.5	6	57.3	0.820	1.455	0.0004	0.0007
148	Crackers (Vegetable)	G-367	MALAYSIA	0.5	15	64.6	0.412	0.676	0.0002	0.0003
149	Crackers (Seafood)	-	INDONESIA	0.5	1	65.0	0.142	0.142	0.0001	0.0001
150	Crackers (Vegetable)	F-60	SINGAPORE	172.9	15	64.6	0.412	0.676	0.0712	0.1169

151	Dried snack titbits (Fish)	D-106	MALAYSIA	0.5	1	65.0	0.142	0.142	0.0001	0.0001
152	Dried snack titbits (Fish)	D-22	JAPAN	93.4	15	64.6	0.412	0.676	0.0384	0.0632
153	Dried snack titbits (Fish)	S-103	THAILAND	54.9	1	65.0	0.142	0.142	0.0078	0.0078
154	Dried snack titbits (Cuttlefish)	-	SINGAPORE	32.4	1	65.0	0.142	0.142	0.0046	0.0046
155	Dried snack titbits (Fish)	D-106	MALAYSIA	21.1	1	65.0	0.142	0.142	0.0030	0.0030
156	Dried snack titbits (Fish)	O-364	JAPAN	17.2	15	64.6	0.412	0.676	0.0071	0.0116
157	Dried snack titbits (Cuttlefish)	Y-30	Unknown	14.2	1	65.0	0.142	0.142	0.0020	0.0020
158	Dried snack titbits (Fish)	S-256	MALAYSIA	13	1	65.0	0.142	0.142	0.0018	0.0018
159	Dried snack titbits (Cuttlefish)	E-399	SINGAPORE	10.6	1	65.0	0.142	0.142	0.0015	0.0015
160	Dried snack titbits (Cuttlefish)	K-89	VIETNAM	0.5	1	65.0	0.142	0.142	0.0001	0.0001
161	Dried snack titbits (Cuttlefish)	T-311	SINGAPORE	0.5	1	65.0	0.142	0.142	0.0001	0.0001
162	Dried snack titbits (Fish)	K-89	THAILAND	0.5	1	65.0	0.142	0.142	0.0001	0.0001

163	Dried snack titbits (Fish)	D-106	MALAYSIA	0.5	1	65.0	0.142	0.142	0.0001	0.0001
164	Dried snack titbits (Fish)	D-106	MALAYSIA	0.5	1	65.0	0.142	0.142	0.0001	0.0001
165	Dried snack titbits (Cuttlefish)	S-37	SINGAPORE	0.5	1	65.0	0.142	0.142	0.0001	0.0001
166	Dried snack titbits (Fish)	D-106	MALAYSIA	0.5	1	65.0	0.142	0.142	0.0001	0.0001
167	Dried snack titbits (Fish)	S-256	MALAYSIA	0.5	1	65.0	0.142	0.142	0.0001	0.0001
168	Dried snack titbits (Fish)	D-106	MALAYSIA	0.5	1	65.0	0.142	0.142	0.0001	0.0001
169	Dried snack titbits (Cuttlefish)	L-268	SINGAPORE	0.5	1	65.0	0.142	0.142	0.0001	0.0001
170	Dried snack titbits (Cuttlefish)	B-478	THAILAND	0.5	1	65.0	0.142	0.142	0.0001	0.0001
171	Dried snack titbits (Cuttlefish)	K-89	VIETNAM	0.5	1	65.0	0.142	0.142	0.0001	0.0001
172	Dried snack titbits (Cuttlefish)	K-89	VIETNAM	0.5	1	65.0	0.142	0.142	0.0001	0.0001
173	Dried snack titbits (Cuttlefish)	S-37	SINGAPORE	11.4	1	65.0	0.142	0.142	0.0016	0.0016
174	Dried snack titbits (Cuttlefish)	K-89	VIETNAM	20.4	1	65.0	0.142	0.142	0.0029	0.0029

175	Dried snack titbits (Cuttlefish)	B-478	THAILAND	26.9	1	65.0	0.142	0.142	0.0038	0.0038
176	Dried snack titbits (Cuttlefish)	-	SINGAPORE	37.4	1	65.0	0.142	0.142	0.0053	0.0053
177	Dried snack titbits (Squid)	H-132	JAPAN	0.5	1	65.0	0.142	0.142	0.0001	0.0001
178	Dried snack titbits (Squid)	I-432	JAPAN	0.5	1	65.0	0.142	0.142	0.0001	0.0001
179	Dried snack titbits (Squid)	H-128	JAPAN	0.5	1	65.0	0.142	0.142	0.0001	0.0001
180	Dried snack titbits (Squid)	-	JAPAN	0.5	1	65.0	0.142	0.142	0.0001	0.0001
181	Dried snack titbits (Squid)	G-159	THAILAND	0.5	1	65.0	0.142	0.142	0.0001	0.0001
182	Dried snack titbits (Cuttlefish)	D-438	SINGAPORE	0.5	1	65.0	0.142	0.142	0.0001	0.0001
183	Dried snack titbits (Squid)	B-478	THAILAND	0.5	1	65.0	0.142	0.142	0.0001	0.0001
184	Dried snack titbits (Cuttlefish)	K-89	VIETNAM	39	1	65.0	0.142	0.142	0.0055	0.0055
185	Biscuit (Unsweetened)	M-240	UNITED KINGDOM	673.7	54	58.6	0.451	0.935	0.3035	0.6298
186	Biscuit (Unsweetened)	M-179	MALAYSIA	665.3	54	58.6	0.451	0.935	0.2998	0.6220

187	Biscuit (Unsweetened)	H-351	MALAYSIA	578.9	54	58.6	0.451	0.935	0.2608	0.5412
188	Biscuit (Unsweetened)	M-264	UNITED KINGDOM	559.4	54	58.6	0.451	0.935	0.2520	0.5230
189	Biscuit (Sweetened)	-	SINGAPORE	510.2	54	58.6	0.451	0.935	0.2299	0.4770
190	Biscuit (Sweetened)	J-349	MALAYSIA	509.8	54	58.6	0.451	0.935	0.2297	0.4766
191	Biscuit (Unsweetened)	H-351	MALAYSIA	462.4	54	58.6	0.451	0.935	0.2083	0.4323
192	Biscuit (Sweetened)	K-191	SINGAPORE	436.8	54	58.6	0.451	0.935	0.1968	0.4084
193	Biscuit (unspecified)	H-351	MALAYSIA	436.7	54	58.6	0.451	0.935	0.1968	0.4083
194	Biscuit (Sweetened)	H-351	MALAYSIA	423.1	54	58.6	0.451	0.935	0.1906	0.3955
195	Biscuit (Sweetened)	M-212	UNITED KINGDOM	421.9	54	58.6	0.451	0.935	0.1901	0.3944
196	Biscuit (Unsweetened)	J-349	MALAYSIA	419.8	54	58.6	0.451	0.935	0.1891	0.3925
197	Biscuit (Unsweetened)	P-447	MALAYSIA	418.4	54	58.6	0.451	0.935	0.1885	0.3911
198	Biscuit (unspecified)	A-69	AUSTRALIA	404.7	54	58.6	0.451	0.935	0.1823	0.3783
199	Biscuit (unspecified)	K-191	MALAYSIA	391	54	58.6	0.451	0.935	0.1762	0.3655

200	Biscuit (unspecified)	M-264	UNITED KINGDOM	372.2	54	58.6	0.451	0.935	0.1677	0.3480
201	Biscuit (Unsweetened)	M-264	UNITED KINGDOM	362.5	54	58.6	0.451	0.935	0.1633	0.3389
202	Biscuit (Sweetened)	S-0	UNITED KINGDOM	344.9	54	58.6	0.451	0.935	0.1554	0.3224
203	Biscuit (Sweetened)	T-352	INDONESIA	331.6	54	58.6	0.451	0.935	0.1494	0.3100
204	Biscuit (Unsweetened)	S-89	MALAYSIA	324.5	54	58.6	0.451	0.935	0.1462	0.3034
205	Biscuit (unspecified)	T-204	UNITED KINGDOM	324.3	54	58.6	0.451	0.935	0.1461	0.3032
206	Biscuit (Sweetened)	J-349	MALAYSIA	295.9	54	58.6	0.451	0.935	0.1333	0.2766
207	Biscuit (Unsweetened)	C-478	MALAYSIA	294.9	54	58.6	0.451	0.935	0.1329	0.2757
208	Biscuit (Sweetened)	K-191	SINGAPORE	293.1	54	58.6	0.451	0.935	0.1321	0.2740
209	Biscuit (Sweetened)	B-425	INDIA	290.8	54	58.6	0.451	0.935	0.1310	0.2719
210	Biscuit (Sweetened)	A-69	AUSTRALIA	289.9	54	58.6	0.451	0.935	0.1306	0.2710
211	Biscuit (Unsweetened)	O-183	TAIWAN	283.7	54	58.6	0.451	0.935	0.1278	0.2652
212	Biscuit (Sweetened)	L-469	MALAYSIA	283.1	54	58.6	0.451	0.935	0.1276	0.2647
213	Biscuit (Sweetened)	H-351	MALAYSIA	281.3	54	58.6	0.451	0.935	0.1267	0.2630

214	Biscuit (Sweetened)	M-240	UNITED KINGDOM	266.9	54	58.6	0.451	0.935	0.1203	0.2495
215	Biscuit (unspecified)	T-204	UNITED KINGDOM	260.2	54	58.6	0.451	0.935	0.1172	0.2433
216	Biscuit (Sweetened)	M-240	UNITED KINGDOM	254.7	54	58.6	0.451	0.935	0.1148	0.2381
217	Biscuit (unspecified)	T-204	UNITED KINGDOM	251.6	54	58.6	0.451	0.935	0.1134	0.2352
218	Biscuit (Sweetened)	M-264	UNITED KINGDOM	239.8	54	58.6	0.451	0.935	0.1080	0.2242
219	Biscuit (Sweetened)	C-321	FRANCE	237.7	54	58.6	0.451	0.935	0.1071	0.2222
220	Biscuit (unspecified)	T-352	MALAYSIA	234.3	54	58.6	0.451	0.935	0.1056	0.2190
221	Biscuit (Sweetened)	F-60	MALAYSIA	234.3	54	58.6	0.451	0.935	0.1056	0.2190
222	Biscuit (unspecified)	S-89	MALAYSIA	228.2	54	58.6	0.451	0.935	0.1028	0.2133
223	Biscuit (Sweetened)	S-63	MALAYSIA	201	54	58.6	0.451	0.935	0.0906	0.1879
224	Biscuit (Sweetened)	T-352	INDONESIA	198.8	54	58.6	0.451	0.935	0.0896	0.1859
225	Biscuit (Unsweetened)	K-196	MALAYSIA	195.2	54	58.6	0.451	0.935	0.0880	0.1825
226	Biscuit (Sweetened)	J-349	MALAYSIA	193.5	54	58.6	0.451	0.935	0.0872	0.1809
227	Biscuit (Sweetened)	W-210	SINGAPORE	181.1	54	58.6	0.451	0.935	0.0816	0.1693

228	Biscuit (Unsweetened)	C-177	MALAYSIA	173.7	54	58.6	0.451	0.935	0.0783	0.1624
229	Biscuit (unspecified)	M-264	UNITED KINGDOM	167.5	54	58.6	0.451	0.935	0.0755	0.1566
230	Biscuit (unspecified)	F-60	MALAYSIA	158.7	54	58.6	0.451	0.935	0.0715	0.1484
231	Biscuit (unspecified)	F-60	MALAYSIA	158.6	54	58.6	0.451	0.935	0.0715	0.1483
232	Biscuit (unspecified)	O-67	INDONESIA	158.4	54	58.6	0.451	0.935	0.0714	0.1481
233	Biscuit (Unsweetened)	L-469	SINGAPORE	156.2	54	58.6	0.451	0.935	0.0704	0.1460
234	Biscuit (Sweetened)	K-191	SINGAPORE	154.1	54	58.6	0.451	0.935	0.0694	0.1441
235	Biscuit (Sweetened)	-	MALAYSIA	151.3	54	58.6	0.451	0.935	0.0682	0.1414
236	Biscuit (Sweetened)	M-179	MALAYSIA	150.6	54	58.6	0.451	0.935	0.0679	0.1408
237	Biscuit (Unsweetened)	C-66	MALAYSIA	148.1	54	58.6	0.451	0.935	0.0667	0.1385
238	Biscuit (Unsweetened)	J-349	SINGAPORE	142.3	54	58.6	0.451	0.935	0.0641	0.1330
239	Biscuit (unspecified)	S-321	CHINA	140.2	54	58.6	0.451	0.935	0.0632	0.1311
240	Biscuit (Sweetened)	N-59	JAPAN	129.7	54	58.6	0.451	0.935	0.0584	0.1213

241	Biscuit (Unsweetened)	L-483	MALAYSIA	128.2	54	58.6	0.451	0.935	0.0578	0.1199
242	Biscuit (Sweetened)	N-317	COLOMBIA	125.9	54	58.6	0.451	0.935	0.0567	0.1177
243	Wafer	J-365	BELGIUM	125.4	8	60.0	0.876	1.279	0.1099	0.1604
244	Biscuit (Sweetened)	C-75	MALAYSIA	107.8	54	58.6	0.451	0.935	0.0486	0.1008
245	Wafer	A-131	MALAYSIA	105.9	8	60.0	0.876	1.279	0.0928	0.1355
246	Biscuit (unspecified)	T-204	UNITED KINGDOM	104.3	54	58.6	0.451	0.935	0.0470	0.0975
247	Biscuit (unspecified)	V-104	CANADA	102.6	54	58.6	0.451	0.935	0.0462	0.0959
248	Biscuit (unspecified)	B-128	GERMANY	102	54	58.6	0.451	0.935	0.0460	0.0954
249	Biscuit (unspecified)	A-69	AUSTRALIA	101.4	54	58.6	0.451	0.935	0.0457	0.0948
250	Biscuit (Unsweetened)	J-314	THAILAND	100.1	54	58.6	0.451	0.935	0.0451	0.0936
251	Biscuit (Sweetened)	-	SINGAPORE	99.7	54	58.6	0.451	0.935	0.0449	0.0932
252	Biscuit (Sweetened)	-	MALAYSIA	94.2	54	58.6	0.451	0.935	0.0424	0.0881
253	Biscuit (Unsweetened)	K-196	MALAYSIA	87.2	54	58.6	0.451	0.935	0.0393	0.0815
254	Biscuit (Sweetened)	B-128	GERMANY	85.7	54	58.6	0.451	0.935	0.0386	0.0801

255	Biscuit (unspecified)	M-492	MALAYSIA	84.1	54	58.6	0.451	0.935	0.0379	0.0786
256	Wafer	-	SINGAPORE	78.9	8	60.0	0.876	1.279	0.0691	0.1009
257	Biscuit (unspecified)	B-128	GERMANY	74.2	54	58.6	0.451	0.935	0.0334	0.0694
258	Biscuit (Unsweetened)	J-74	MALAYSIA	73.6	54	58.6	0.451	0.935	0.0332	0.0688
259	Biscuit (unspecified)	B-128	GERMANY	60.3	54	58.6	0.451	0.935	0.0272	0.0564
260	Biscuit (unspecified)	-	MALAYSIA	46.7	54	58.6	0.451	0.935	0.0210	0.0437
261	Wafer	L-215	AUSTRIA	46.5	8	60.0	0.876	1.279	0.0407	0.0595
262	Biscuit (Sweetened)	M-30	NETHERLANDS	37.9	54	58.6	0.451	0.935	0.0171	0.0354
263	Wafer	R-285	VIETNAM	29.8	8	60.0	0.876	1.279	0.0261	0.0381
264	Biscuit (unspecified)	C-326	SINGAPORE	26.4	54	58.6	0.451	0.935	0.0119	0.0247
265	Biscuit (Sweetened)	L-215	AUSTRIA	22.3	54	58.6	0.451	0.935	0.0100	0.0208
266	Biscuit (unspecified)	C-73	SINGAPORE	20.5	54	58.6	0.451	0.935	0.0092	0.0192
267	Biscuit (unspecified)	M-397	NETHERLANDS	16.5	54	58.6	0.451	0.935	0.0074	0.0154

268	Biscuit (Unsweetened)	M-409	SINGAPORE	15.8	54	58.6	0.451	0.935	0.0071	0.0148
269	Wafer	R-116	INDONESIA	13.2	8	60.0	0.876	1.279	0.0116	0.0169
270	Wafer	G-365	HONG KONG	12.7	8	60.0	0.876	1.279	0.0111	0.0162
271	Wafer	G-365	HONG KONG	11.4	8	60.0	0.876	1.279	0.0100	0.0146
272	Biscuit (unspecified)	L-294	BELGIUM	11.1	54	58.6	0.451	0.935	0.0050	0.0104
273	Biscuit (unspecified)	B-359	CHINA	0.5	54	58.6	0.451	0.935	0.0002	0.0005
274	Biscuit (Sweetened)	D-93	SINGAPORE	0.5	54	58.6	0.451	0.935	0.0002	0.0005
275	Biscuit (unspecified)	L-215	AUSTRIA	0.5	54	58.6	0.451	0.935	0.0002	0.0005
276	Wafer	S-103	VIETNAM	0.5	8	60.0	0.876	1.279	0.0004	0.0006
277	Wafer	R-116	INDONESIA	0.5	8	60.0	0.876	1.279	0.0004	0.0006
278	Wafer	B-391	VIETNAM	0.5	8	60.0	0.876	1.279	0.0004	0.0006
279	Wafer	K-294	UNITED STATES	0.5	8	60.0	0.876	1.279	0.0004	0.0006
280	Biscuit (Unsweetened)	H-351	MALAYSIA	135.7	54	58.6	0.451	0.935	0.0611	0.1269
281	Biscuit (Unsweetened)	S-237	SINGAPORE	39.3	54	58.6	0.451	0.935	0.0177	0.0367
282	Wafer	M-218	VIETNAM	0.5	8	60.0	0.876	1.279	0.0004	0.0006

283	Cereal bar (Whole Grain)	N-91	UNITED STATES	353.1	109	61.5	0.330	0.748	0.1167	0.2643
284	Cereal bar (Whole rolled)	S-410	UNITED STATES	239.3	109	61.5	0.330	0.748	0.0791	0.1791
285	Popcorn	U-92	VIETNAM	143.6	109	61.5	0.330	0.748	0.0474	0.1075
286	Cereal bar (Whole Grain)	A-245	UNITED KINGDOM	59.8	109	61.5	0.330	0.748	0.0198	0.0448
287	Cereal bar (Whole Grain)	A-245	UNITED KINGDOM	47.9	109	61.5	0.330	0.748	0.0158	0.0359
288	Cereal bar (Whole Grain)	N-317	AUSTRALIA	40	109	61.5	0.330	0.748	0.0132	0.0299
289	Cereal bar (Whole Grain)	N-317	POLAND	13.9	109	61.5	0.330	0.748	0.0046	0.0104
290	Popcorn	F-60	SINGAPORE	128.4	109	61.5	0.330	0.748	0.0424	0.0961
291	Popcorn	N-213	CANADA	89.1	109	61.5	0.330	0.748	0.0294	0.0667
292	Popcorn	F-60	SINGAPORE	182.3	109	61.5	0.330	0.748	0.0602	0.1364
293	Popcorn	N-213	CANADA	50.2	109	61.5	0.330	0.748	0.0166	0.0376
294	Cereal bar (Oat flakes)	Y-406	MALAYSIA	53.8	109	61.5	0.330	0.748	0.0178	0.0403
295	Cereal bar (Oat flakes)	C-321	FRANCE	0.5	109	61.5	0.330	0.748	0.0002	0.0004

Table S21: Summary of results for beverages (coffee and coffee substitutes) (n=51)

No.	Food Products and Information			LC-MS/MS Analysis of Acrylamide	24-hour Dietary Recall Survey on Consumers (eaters only) from aged 15 to 92 years old					
	Food Product	Brand name (coded)	Country of origin	Concentration (µg/kg)	Number of consumers surveyed	Mean Body Weight, (kg bw)	Consumption of mean consumers (g/kg bw)	Consumption of high consumers (P95) (g/kg bw)	Dietary exposure of mean consumers (µg/kg bw/day)	Dietary exposure of high consumers (P95) (µg/kg bw/day)
1	Coffee powder (Roasted)	N-211	INDONESIA	593.3	219	68.5	2.032	3.428	0.0161	0.0271
2	Coffee powder (Roasted)	-	SINGAPORE	251.2	219	68.5	2.032	3.428	0.0173	0.0291
3	Coffee powder (Roasted)	B-222	SINGAPORE	234.9	219	68.5	2.032	3.428	0.0267	0.0451
4	Coffee powder (Roasted)	O-242	SINGAPORE	207.7	219	68.5	2.032	3.428	0.0236	0.0399
5	Coffee powder (Roasted)	K-401	SINGAPORE	196.7	219	68.5	2.032	3.428	0.0224	0.0378
6	Coffee powder (Roasted)	-	SINGAPORE	187.4	219	68.5	2.032	3.428	0.0213	0.0360
7	Coffee powder (Roasted)	C-350	SINGAPORE	175.8	219	68.5	2.032	3.428	0.0214	0.0362

8	Coffee powder (Roasted)	C-456	SINGAPORE	174.6	219	68.5	2.032	3.428	0.0199	0.0335
9	Coffee powder (Roasted)	G-45	SINGAPORE	171.1	219	68.5	2.032	3.428	0.0195	0.0328
10	Coffee powder (Roasted)	S-270	SINGAPORE	162.6	219	68.5	2.032	3.428	0.0185	0.0312
11	Coffee powder (Roasted)	-	SINGAPORE	147.1	219	68.5	2.032	3.428	0.0167	0.0282
12	Coffee powder (Roasted)	H-293	SINGAPORE	137.5	219	68.5	2.032	3.428	0.0391	0.0660
13	Coffee powder (Roasted)	-	SINGAPORE	109	219	68.5	2.032	3.428	0.0124	0.0209
14	Coffee powder (Roasted)	S-417	SINGAPORE	96.5	219	68.5	2.032	3.428	0.0110	0.0185
15	Coffee powder (Roasted)	Y-382	SINGAPORE	93.3	219	68.5	2.032	3.428	0.0106	0.0179
16	Coffee powder (Roasted)	S-28	SINGAPORE	85.4	219	68.5	2.032	3.428	0.0097	0.0164
17	Coffee powder (Roasted)	C-471	SINGAPORE	83.5	219	68.5	2.032	3.428	0.0095	0.0160
18	Coffee powder (Roasted)	-	SINGAPORE	83.5	219	68.5	2.032	3.428	0.0095	0.0160
19	Coffee powder (Roasted)	-	SINGAPORE	80.3	219	68.5	2.032	3.428	0.0091	0.0154

20	Coffee powder (Roasted)	-	SINGAPORE	79.7	219	68.5	2.032	3.428	0.0091	0.0153
21	Coffee powder (Roasted)	O-187	MALAYSIA	76.4	219	68.5	2.032	3.428	0.0072	0.0122
22	Coffee powder (Roasted)	C-234	SINGAPORE	65.4	219	68.5	2.032	3.428	0.0074	0.0126
23	Coffee powder (Roasted)	-	MALAYSIA	59.9	219	68.5	2.032	3.428	0.0068	0.0115
24	Coffee powder (Roasted)	D-396	SINGAPORE	51.3	219	68.5	2.032	3.428	0.0058	0.0098
25	Coffee powder (Roasted)	K-253	SINGAPORE	45.8	219	68.5	2.032	3.428	0.0052	0.0088
26	Coffee powder (Instant)	S-188	SINGAPORE	102.1	70	62.1	1.187	2.991	0.0170	0.0428
27	Coffee powder (Instant)	C-320	SINGAPORE	70.9	70	62.1	1.187	2.991	0.0067	0.0170
28	Coffee powder (Instant)	G-273	SINGAPORE	65.6	70	62.1	1.187	2.991	0.0062	0.0157
29	Coffee powder (Instant)	R-277	MALAYSIA	49.1	70	62.1	1.187	2.991	0.0117	0.0294
30	Coffee powder (Instant)	N-211	SINGAPORE	48.8	70	62.1	1.187	2.991	0.0061	0.0154
31	Coffee powder (Instant)	M-263	MALAYSIA	25.7	70	62.1	1.187	2.991	0.0041	0.0102

32	Cereal powder (Malted)	H-372	INDIA	144.5	70	62.1	1.187	2.991	0.0303	0.0763
33	Cereal powder (Malted)	H-372	INDIA	127.6	70	62.1	1.187	2.991	0.0267	0.0674
34	Cereal powder (Malted)	C-258	INDIA	83.1	70	62.1	1.187	2.991	0.0174	0.0439
35	Cereal powder (Malted)	N-72	MALAYSIA	65.6	70	62.1	1.187	2.991	0.0137	0.0346
36	Cereal powder (Malted)	O-233	THAILAND	50.5	70	62.1	1.187	2.991	0.0106	0.0267
37	Cereal powder (Malted)	H-372	MALAYSIA	34.2	70	62.1	1.187	2.991	0.0072	0.0181
38	Cereal powder (Malted)	O-233	THAILAND	31.7	70	62.1	1.187	2.991	0.0018	0.0046
39	Cereal powder (Malted)	N-317	SINGAPORE	23.3	70	62.1	1.187	2.991	0.0049	0.0123
40	Cereal powder (Malted)	H-372	MALAYSIA	22.7	70	62.1	1.187	2.991	0.0048	0.0120
41	Cereal powder (Malted)	H-372	MALAYSIA	17.4	70	62.1	1.187	2.991	0.0036	0.0092
42	Cereal powder (Malted)	O-233	MALAYSIA	10.1	70	62.1	1.187	2.991	0.0006	0.0015
43	Cereal powder (Malted)	N-317	SINGAPORE	90	70	62.1	1.187	2.991	0.0189	0.0475

44	Cereal powder (Malted)	O-233	THAILAND	68	70	62.1	1.187	2.991	0.0039	0.0099
45	Cereal powder (Malted)	H-372	MALAYSIA	104	70	62.1	1.187	2.991	0.0218	0.0549
46	Cereal powder (Malted)	H-372	MALAYSIA	84	70	62.1	1.187	2.991	0.0176	0.0443
47	Cereal powder (Malted)	N-317	MALAYSIA	6	70	62.1	1.187	2.991	0.0013	0.0032
48	Cereal powder (Malted)	H-372	MALAYSIA	87	70	62.1	1.187	2.991	0.0182	0.0459
49	Cereal powder (Malted)	O-233	THAILAND	212	70	62.1	1.187	2.991	0.0122	0.0307
50	Cereal powder (Malted)	O-233	THAILAND	85	70	62.1	1.187	2.991	0.0049	0.0123
51	Cereal powder (Malted)	V-330	MALAYSIA	170	70	62.1	1.187	2.991	0.0356	0.0897

Table S22: Summary of results for nuts (n=40)

	Food Products and Information	LC-MS/MS Analysis of Acrylamide	24-hour Dietary Recall Survey on Consumers (eaters only) from aged 15 to 92 years old

No.	Food Product	Brand name (coded)	Country of origin	Concentration (µg/kg)	Number of consumers surveyed	Mean Body Weight, (kg bw)	Consumption of mean consumers (g/kg bw)	Consumption of high consumers (P95) (g/kg bw)	Dietary exposure of mean consumers (µg/kg bw/day)	Dietary exposure of high consumers (P95) (µg/kg bw/day)
1	Nuts (Mixed)	T-214	SINGAPORE	247.2	33	64.5	0.157	0.476	0.0388	0.1177
2	Nuts (Peanuts)	H-13	CHINA	188.5	130	64.8	0.188	0.827	0.0354	0.1559
3	Nuts (Mixed)	F-60	Unknown	96.4	33	64.5	0.157	0.476	0.0151	0.0459
4	Nuts (Mixed)	T-489	MALAYSIA	84.3	33	64.5	0.157	0.476	0.0132	0.0401
5	Nuts (Peanuts)	T-379	MALAYSIA	76.1	130	64.8	0.188	0.827	0.0143	0.0630
6	Nuts (Cashew)	G-227	TAIWAN	72	39	62.6	0.287	1.379	0.0207	0.0993
7	Nuts (Almond)	C-375	SINGAPORE	71.3	37	60.4	0.175	0.594	0.0124	0.0423
8	Nuts (Mixed)	M-240	HONG KONG	68.7	33	64.5	0.157	0.476	0.0108	0.0327
9	Nuts (Almond)	F-60	MALAYSIA	63.1	37	60.4	0.175	0.594	0.0110	0.0375
10	Nuts (Peanuts)	C-375	SINGAPORE	59.8	130	64.8	0.188	0.827	0.0112	0.0495
11	Nuts (Mixed)	T-44	MALAYSIA	57.7	33	64.5	0.157	0.476	0.0091	0.0275
12	Nuts (Mixed)	C-375	SINGAPORE	57.1	33	64.5	0.157	0.476	0.0090	0.0272
13	Nuts (Mixed)	E-399	TAIWAN	56.8	33	64.5	0.157	0.476	0.0089	0.0271
14	Nuts (Cashew)	M-240	MALAYSIA	50.2	39	62.6	0.287	1.379	0.0144	0.0692

15	Nuts (Peanuts)	F-60	MALAYSIA	49.4	130	64.8	0.188	0.827	0.0093	0.0409
16	Nuts (Mixed)	S-431	THAILAND	48.9	33	64.5	0.157	0.476	0.0077	0.0233
17	Nuts (Walnut)	M-240	TAIWAN	38.8	28	61.3	0.143	0.632	0.0055	0.0245
18	Nuts (Peanuts)	C-375	SINGAPORE	35.2	130	64.8	0.188	0.827	0.0066	0.0291
19	Nuts (Mixed)	T-44	MALAYSIA	35	33	64.5	0.157	0.476	0.0055	0.0167
20	Nuts (Peanuts)	B-496	MALAYSIA	33.7	130	64.8	0.188	0.827	0.0063	0.0279
21	Nuts (Peanuts)	D-483	CHINA	32.8	130	64.8	0.188	0.827	0.0062	0.0271
22	Nuts (Cashew)	K-295	THAILAND	13.6	39	62.6	0.287	1.379	0.0039	0.0188
23	Nuts (Cashew)	C-375	SINGAPORE	11.4	39	62.6	0.287	1.379	0.0033	0.0157
24	Nuts (Cashew)	-	SINGAPORE	10.6	39	62.6	0.287	1.379	0.0030	0.0146
25	Nuts (Cashew)	A-347	SINGAPORE	0.5	39	62.6	0.287	1.379	0.0001	0.0007
26	Nuts (Mixed)	N-434	MALAYSIA	0.5	33	64.5	0.157	0.476	0.0001	0.0002
27	Nuts (Brazil nut kernels)	N-438	AUSTRALIA	0.5	2	52.5	0.201	0.336	0.0001	0.0002
28	Nuts (Almond)	H-170	UNITED STATES	0.5	37	60.4	0.175	0.594	0.0001	0.0003
29	Nuts (Peanuts)	F-370	INDONESIA	0.5	130	64.8	0.188	0.827	0.0001	0.0004
30	Nuts (Peanuts)	G-89	MALAYSIA	0.5	130	64.8	0.188	0.827	0.0001	0.0004
31	Nuts (Peanuts)	F-370	INDONESIA	0.5	130	64.8	0.188	0.827	0.0001	0.0004
32	Nuts (Pistachio)	T-44	MALAYSIA	0.5	10	67.6	0.311	0.679	0.0002	0.0003

33	Nuts (Cashew)	S-469	INDIA	0.5	39	62.6	0.287	1.379	0.0001	0.0007
34	Nuts (Almond)	F-190	UNITED STATES	0.5	37	60.4	0.175	0.594	0.0001	0.0003
35	Nuts (Walnut)	G-188	UNITED STATES	0.5	28	61.3	0.143	0.632	0.0001	0.0003
36	Nuts (Peanuts)	T-425	CHINA	0.5	130	64.8	0.188	0.827	0.0001	0.0004
37	Nuts (Chestnuts)	G-39	CHINA	77.9	33	64.5	0.157	0.476	0.0122	0.0371
38	Nuts (Peanuts)	L-322	CHINA	0.5	130	64.8	0.188	0.827	0.0001	0.0004
39	Nuts (Chestnuts)	S-11	CHINA	14	33	64.5	0.157	0.476	0.0022	0.0067
40	Nuts (Peanuts)	-	CHINA	0.5	130	64.8	0.188	0.827	0.0001	0.0004

Contribution of the different food groups to dietary acrylamide exposures for the general and high consumers in Singapore

Figure S1 showed the individual food contributors to the overall dietary exposure of acrylamide from general consumers consumed within and outside the main meals. The largest contributor at 44.8% was ready-to-eat savouries such as crackers and chips, biscuits and wafers, cereal-based snacks and dried snack foods consumed outside the main meals. The second largest contributor at 12.9% was from cooked vegetables such as brassica vegetables, fruiting vegetables, leafy vegetables and herbs, legumes, stalk, stem and bulb vegetables, root and tubers, fungi and seaweed consumed within the main meals.

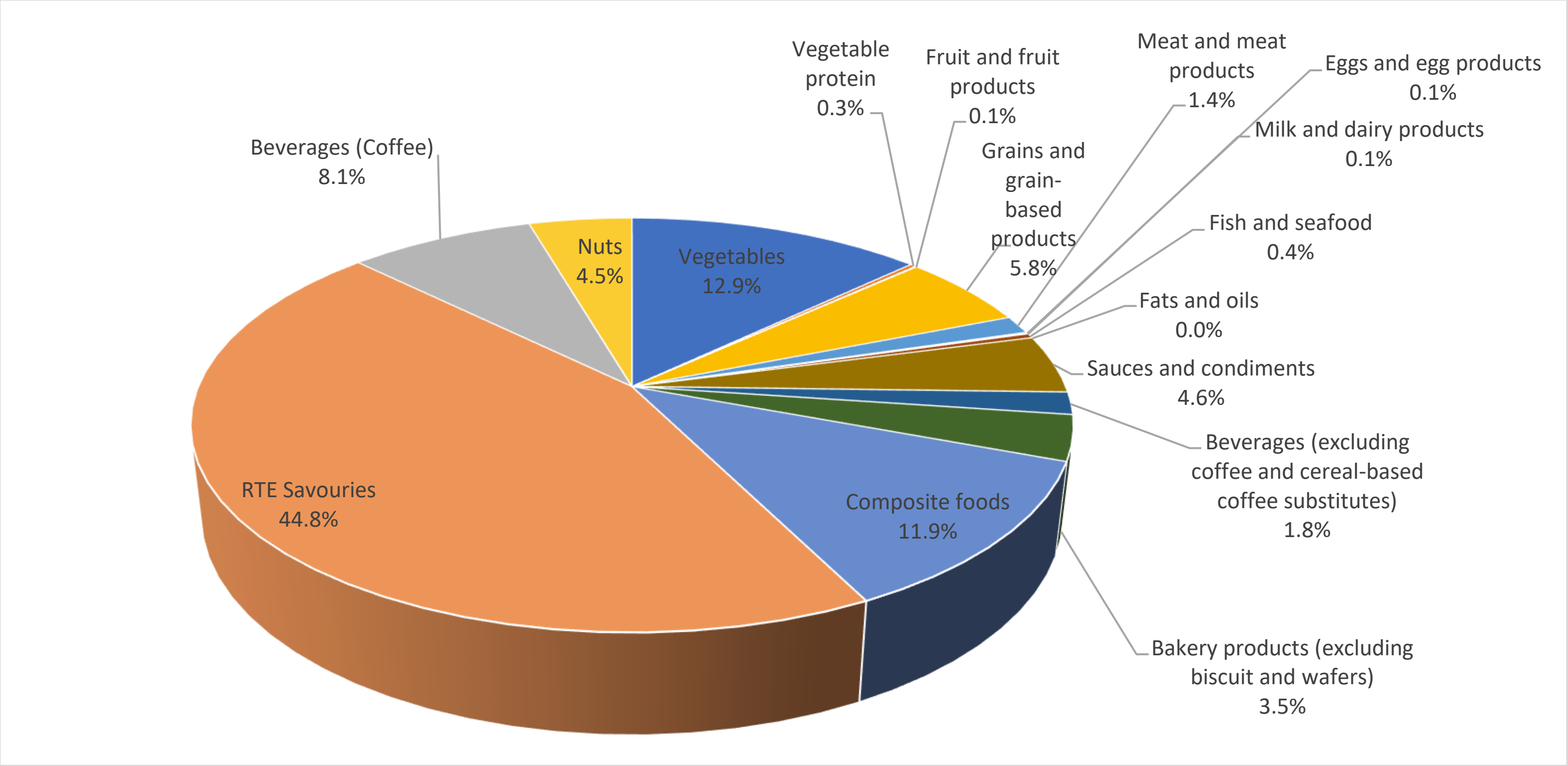

Figure S1: Contribution of individual food category from within and outside of main meals to the overall dietary total of acrylamide for general consumers.

Figure S2 showed the individual food contributors to the overall dietary exposure of acrylamide from high consumers (95th percentile) consumed within and outside the main meals. The largest contributor at 40.4% was ready-to-eat savouries such as crackers and chips, biscuits and wafers, cereal-based snacks and dried snack foods consumed outside the main meals. The second largest contributor at 16.7% was from cooked vegetables such as brassica vegetables, fruiting vegetables, leafy vegetables and herbs, legumes, stalk, stem and bulb vegetables, root and tubers, fungi and seaweed consumed within the main meals

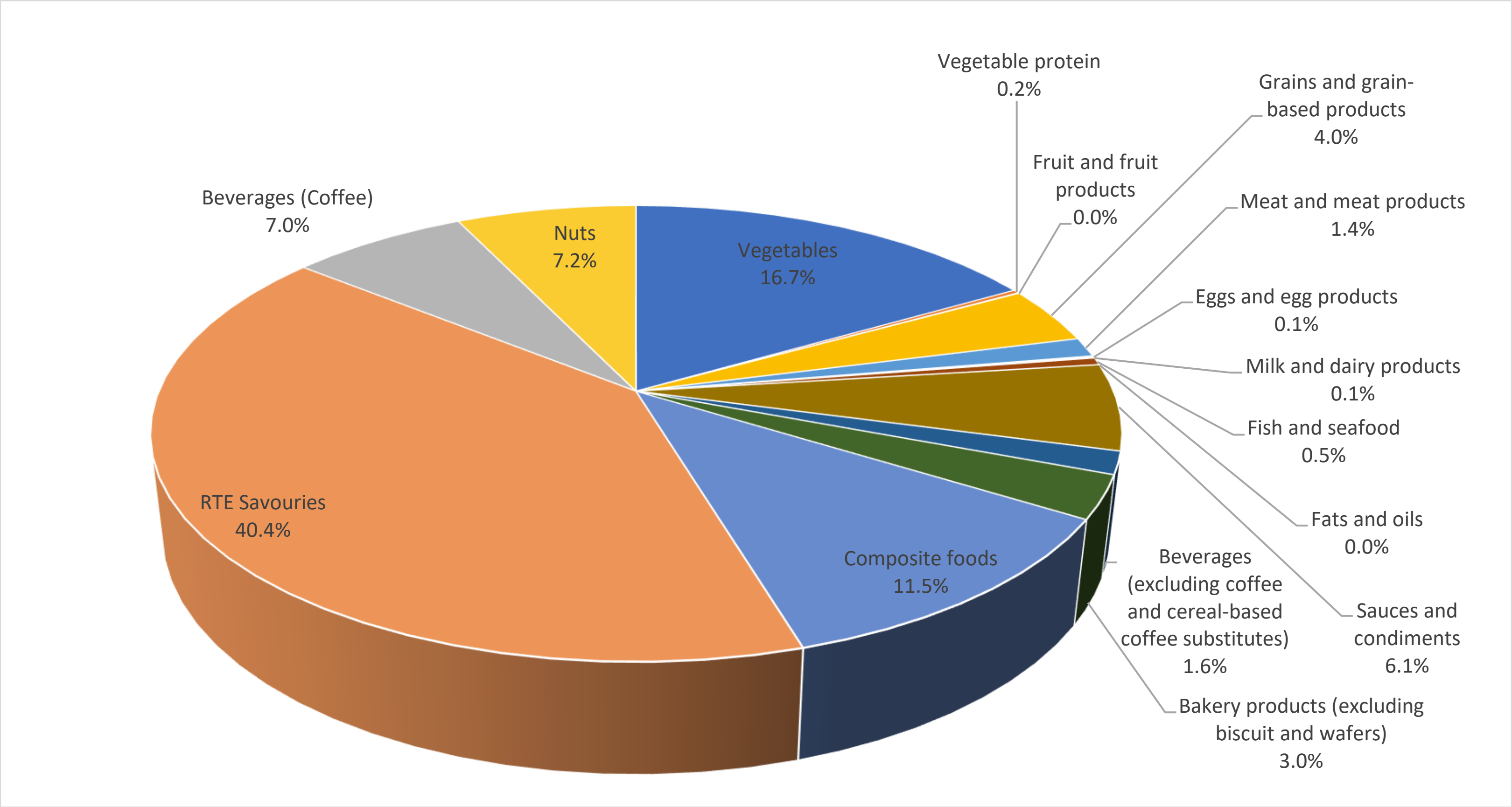

Figure S2: Contribution of individual food category from within and outside of main meals to the total dietary exposure of acrylamide for high consumers.
